# EAT1 transcription factor, a non-cell-autonomous regulator of pollen production, activates meiotic small RNA biogenesis in rice anther tapetum

**DOI:** 10.1371/journal.pgen.1007238

**Published:** 2018-02-12

**Authors:** Seijiro Ono, Hua Liu, Katsutoshi Tsuda, Eigo Fukai, Keisuke Tanaka, Takuji Sasaki, Ken-Ichi Nonomura

**Affiliations:** 1 Experimental Farm, National Institute of Genetics, Yata, Mishima, Shizuoka, Japan; 2 Department of Genetics, School of Life Science, The Graduate University for Advanced Studies (SOKENDAI), Yata, Mishima, Shizuoka, Japan; 3 Graduate School of Science and Technology, Niigata University, Ikarashi, Nishi-ku, Niigata, Japan; 4 NODAI Genome Research Center, Tokyo University of Agriculture, Sakuragaoka, Setagaya-ku, Tokyo, Japan; 5 NODAI Research Institute, Tokyo University of Agriculture, Sakuragaoka, Setagaya-ku, Tokyo, Japan; Donald Danforth Plant Science Center, UNITED STATES

## Abstract

The 24-nucleotides (nt) phased secondary small interfering RNA (phasiRNA) is a unique class of plant small RNAs abundantly expressed in monocot anthers at early meiosis. Previously, 44 intergenic regions were identified as the loci for longer precursor RNAs of 24-nt phasiRNAs (24-*PHAS*s) in the rice genome. However, the regulatory mechanism that determines spatiotemporal expression of these RNAs has remained elusive. ETERNAL TAPETUM1 (EAT1) is a basic-helix-loop-helix (bHLH) transcription factor indispensable for induction of programmed cell death (PCD) in postmeiotic anther tapetum, the somatic nursery for pollen production. In this study, EAT1-dependent non-cell-autonomous regulation of male meiosis was evidenced from microscopic observation of the *eat1* mutant, in which meiosis with aberrantly decondensed chromosomes was retarded but accomplished somehow, eventually resulting in abortive microspores due to an aberrant tapetal PCD. EAT1 protein accumulated in tapetal-cell nuclei at early meiosis and postmeiotic microspore stages. Meiotic EAT1 promoted transcription of 24-*PHAS* RNAs at 101 loci, and importantly, also activated *DICER-LIKE5* (*DCL5*, previous *DCL3b* in rice) mRNA transcription that is required for processing of double-stranded 24-*PHAS*s into 24-nt lengths. From the results of the chromatin-immunoprecipitation and transient expression analyses, another tapetum-expressing bHLH protein, TDR INTERACTING PROTEIN2 (TIP2), was suggested to be involved in meiotic small-RNA biogenesis. The transient assay also demonstrated that UNDEVELOPED TAPETUM1 (UDT1)/bHLH164 is a potential interacting partner of both EAT1 and TIP2 during early meiosis. This study indicates that EAT1 is one of key regulators triggering meiotic phasiRNA biogenesis in anther tapetum, and that other bHLH proteins, TIP2 and UDT1, also play some important roles in this process. Spatiotemporal expression control of these bHLH proteins is a clue to orchestrate precise meiosis progression and subsequent pollen production non-cell-autonomously.

## Author summary

Meiotic crossover formation shuffles homologous genes between parental genomes, and enables transmission of new gene sets to the offspring. Frequency and positions of crossovers are determined by numerous genetic and epigenetic factors, and low nucleosome-density regions are associated with crossover hot spots in yeasts and *Arabidopsis*. The epigenetic chromosome landscape is shaped by unevenly distributed modifications of nucleosome components, histones and DNAs. Recently, we found that MEL1 (ARGONAUTE5) promotes large-scale remodeling of meiotic chromosomes with dramatic increases of histone H3 lysine 9 dimethylation, and that loss of MEL1 resulted in early meiotic arrest with few crossovers present. In rice anthers, MEL1-associating small interfering RNAs (masiRNAs) were composed of large amounts of premeiotic 21-nt phasiRNAs, plus low levels of both 24-nt repeat-associated siRNA and meiotic 24-nt phasiRNAs. Production of 24-nt phasiRNA during the meiotic stage was largely EAT1-dependent. Collectively, our findings suggest a possibility that unknown small RNA-mediated signaling regulates male meiosis non-cell-autonomously, probably a downstream output involves large-scale chromosome remodeling promoted by Argonaute proteins, while a possibility of EAT1-dependent, but small RNA-independent signaling cannot be excluded. In any cases, the studies on MEL1 and tapetal bHLH proteins will be a clue to reveal small RNA-mediated processes determining meiotic epigenetic landscape.

## Introduction

Small noncoding RNAs are 20–30 nucleotides (nt) long and associate with Argonaute family proteins to serve as guide molecules for RNA silencing in various biological processes, such as cell type specification, cell proliferation, cell death, metabolic control, transposon silencing and antiviral defense [1]. Plant genomes encode precursors of microRNA (miRNA) and small interfering RNA (siRNA), as do animal genomes [2]. miRNA is produced from a hairpin structure of a single precursor RNA molecule, and siRNA is derived from a precursor RNA that is either naturally double-stranded or is formed by RNA-dependent RNA polymerases.

The third class of animal small RNAs is Piwi-interacting RNA (piRNA). The piRNA is abundantly expressed in the germline and acts in silencing of transposable elements (TEs) [3], massive elimination of paternally derived mRNAs [4], systemic recognition of self and non-self mRNAs [5, 6], and so on. piRNA associates with Piwi family proteins, a distinct subgroup of Argonaute proteins. In contrast, plants have no Piwi family Argonautes [7, 8], and consequently lack piRNA species. In place of piRNA, *trans*-acting siRNA (tasiRNA) and phased secondary siRNA (phasiRNA) are identified as plant-specific small RNA subgroups. In monocot model plants, rice and maize, phasiRNAs are abundantly expressed in the male reproductive organs, and in this study, the term "phasiRNA" will be used for monocot reproductive phasiRNAs derived from protein-noncoding regions. Both tasiRNA and phasiRNA are produced via miRNA-dependent primary processing, and characterized by phased alignment on both sense and antisense strands in genomic regions. However, they are distinct in several points. First, phasiRNAs are abundantly expressed in developing reproductive organs [9–13], while 21-nt tasiRNAs are expressed in both vegetative and reproductive phases [14]. Second, phasiRNAs are transcribed from hundreds or thousands of unique, namely nonrepetitive, intergenic regions [9, 11–13], while a few tasiRNA-producing (*TAS*) loci are conserved in the plant genome [15–17]. Finally, no phasiRNA targetting a protein-coding gene has been identified, whereas tasiRNAs are complementary to particular genes important for defense and developmental events [14]. In plant reproduction, 24-nt unphased siRNAs or 21-nt epigenetically activated siRNAs (easiRNAs) are thought to maintain genome integrity by programmed DNA methylation of TEs [18–20]. The roles of phasiRNAs during plant reproduction largely remain elusive.

In rice, a single-stranded *PHAS* precursor RNA is primarily processed with 22-nt miRNA triggers; miR2118 for 21-*PHAS*s and miR2275 for 24-*PHAS*s [10]. *PHAS* and *TAS* RNA members each have one or two conserved complementary sequences to miRNAs, and are cleaved via the one-hit or two-hit processing pathway; the one-hit mode is mediated by the AGO1-miRNA complex for 5'-end cleavage of precursor RNAs [15] to generate the 3' fragment that becomes double-stranded, and the two-hit mode depends on AGO1- or AGO7-miRNA, which potentially associates with both ends and cleaves either end or both [14]. The processed RNA is made double-stranded by RNA DEPENDENT RNA POLYMERASE6 (RDR6) [21], and chopped into 21- and 24-nt lengths by DICER-LIKE4 (DCL4) and DCL5 (previous DCL3b in rice), respectively [10].

**Fig 1:**
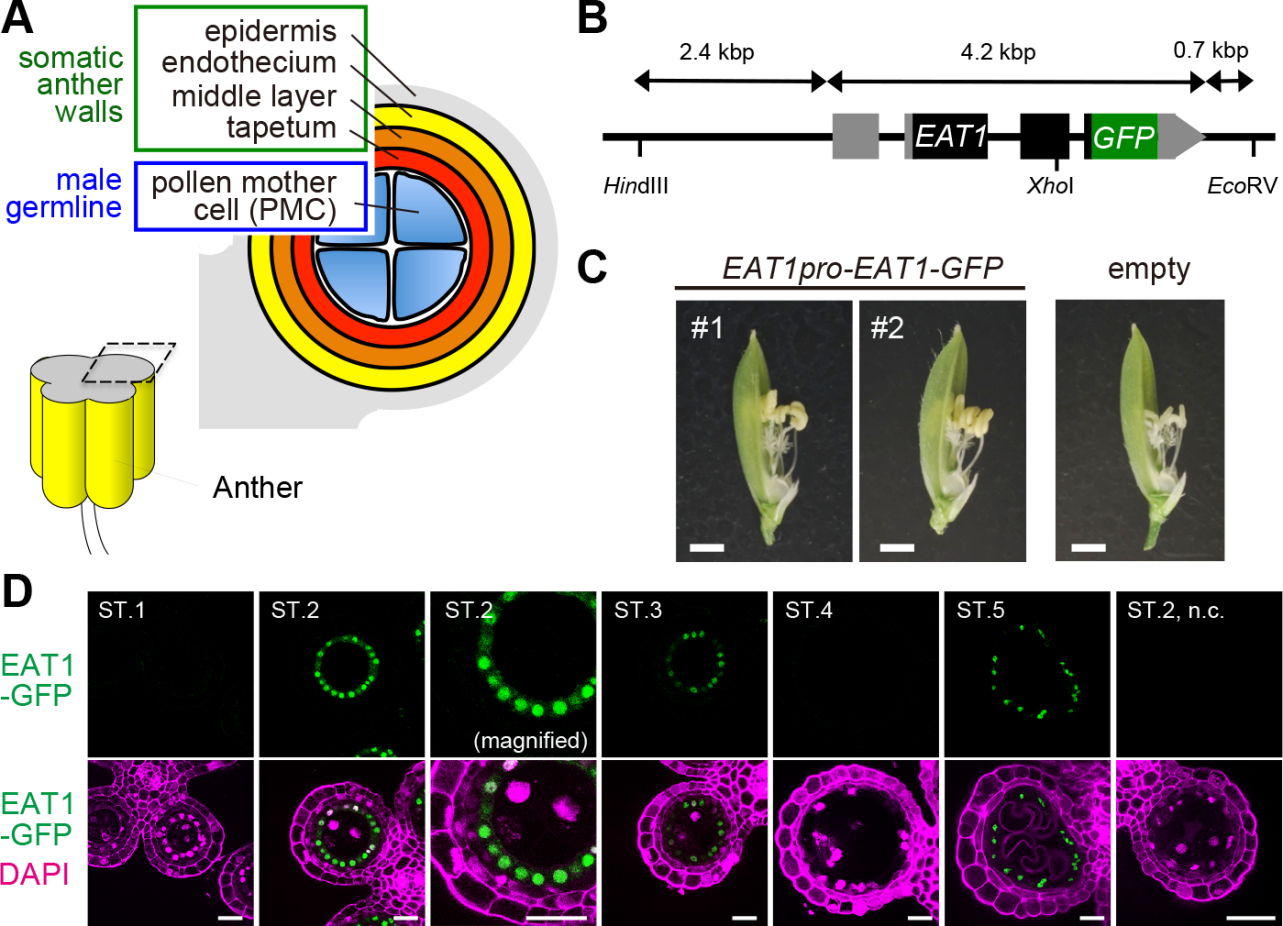
Bimodal expression of EAT1 protein at both early meiosis and postmeiosis in anther tapetum. **(*A*)** Anther lobe architecture around meiosis in rice. **(*B*)** Diagram of the *EAT1*pro-*EAT1-GFP* transcriptional fusion construct. Closed and grey boxes indicate protein coding and untranslated regions, respectively. **(*C*)** *eat1-4*/*eat1-4* flowers of T_0_ plants carrying *EAT1*pro-*EAT1-GFP* (#1, #2) and an empty vector. Bars, 1 mm. Flower images were taken after removal of lemmas. **(*D*)** EAT1-GFP signals (green) in developing anther sections from ST.1 to ST.5. In a transgenic plant harboring the *EAT1*pro-*EAT1-GFP*. The EAT1-GFP signals were restricted to tapetal nuclei in ST.2, ST. 3 and ST.5 anthers, and not detected in the ST.2 anther from the negative control (n.c., right most panel). About the meiotic events and anther lengths corresponding to the respective stages. See Table 1 and S7 Table. Bars, 20 μm.

The anther is a four-lobed male reproductive organ in angiosperms. Each anther lobe is composed of central sporogenous cells and four concentric somatic layers; the epidermis, endothecium, middle layer and tapetum, from outward to inward (Fig 1A) [22–24]. Sporogenous cells undergo several rounds of mitosis and mature into pollen mother cells (PMCs) to prepare for meiosis [22–24]. Maize OUTER CELL LAYER4 (OCL4), an HD-ZIP IV transcription factor (TF), expressed in the anther epidermis and MALE STERILE23 (MS23), a basic helix-loop-helix (bHLH) TF expressed in the tapetum are required for 21 and 24-nt phasiRNA biogenesis, respectively [12, 25]. Small RNA-mediated intercellular signaling is proposed in various steps of plant reproduction, for example, between sperm and vegetative cells in the pollen [19, 26] and between megaspore mother cells and somatic nucellar cells in the ovule [18]. The intercellular movement of reproductive phasiRNAs has been proposed in maize [12, 13], while there is yet no decisive evidence. The underlying mechanism to determine the spatiotemporal expression of reproductive phasiRNAs in anthers has largely remained elusive.

In this study, we focused on the rice bHLH TFs, because they are key transcriptional regulators for differentiation and development of anther somatic layers. TDR INTERACTING PROTEIN2 (TIP2)/bHLH142 is expressed in several undifferentiated cell layers to form the middle layer and tapetum [27, 28]. TAPETUM DEGENERATION RETARDATION (TDR)/bHLH5 makes a heterodimer with TIP2 to promote tapetal differentiation [29]. ETERNAL TAPETUM1 (EAT1)/bHLH141, 41% similar to TIP2, also dimerizes with TDR, and activates transcription of aspartic protease-encoding genes to promote programmed cell death (PCD) of postmeiotic tapetal cells [30, 31]. UNDEVELOPED TAPETUM1 (UDT1)/bHLH164 [32] is expected to function upstream of the regulatory cascade for anther wall development. However, downstream targets of these bHLH TFs are largely unknown.

In addition to its role in tapetal PCD, we found that EAT1 is required earlier in tapetal development to support meiosis, while the loss of EAT1 function has little impact on the tapetum morphology. EAT1 shows a bimodal expression at both early meiosis and postmeiosis. Interestingly, EAT1 expressed during early meiosis promoted both transcription and processing of 24-*PHAS* precursor RNAs to produce 24-nt phasiRNAs in tapetum. This study demonstrates that EAT1 is one of key regulators triggering meiotic phasiRNA biogenesis in anther tapetum, and that other bHLH proteins, TIP2 and UDT1, also play important roles in this process.

## Results

### EAT1 is expressed in anther tapetum during early meiosis

To determine the impact of bHLH proteins in communication between somatic tapetal cells and PMCs in rice anthers, we first performed quantitative reverse-transcription PCR (qRT-PCR) of four *bHLH* genes: *UDT1*, *TDR*, *TIP2* and *EAT1*, all of which are involved in tapetal cell-fate decision [27–32]. In this study, we separated anther developmental processes into six stages to characterize spatiotemporal expression of these genes (ST.1 to ST.6; Table 1). qRT-PCR of meiotic anthers demonstrated that *UDT1*, *TDR* and *TIP2* were expressed as expected from previous reports (S1 Fig, S1 Data). However, *EAT1* expression was bimodal, both at early meiosis (ST.2) and postmeiosis (ST.5), whereas it was previously thought to function only in postmeiotic tapetal PCD [30, 31].

**Table 1:** Developmental stages of rice anthers defined in this study.

Stages	Corresponding germ cell stage ^a^	Corresponding anther wall stage ^a^
ST.1	Premeiotic mitosis	Transition from three- to four-layered
ST.2	Leptotene and zygotene	Undifferentiated tapetum and middle layer
ST.3	Pachytene and diplotene	Tapetum–middle layer differentiation
ST.4	Meiotic division, tetrad	Differentiation completed
ST.5	Microspore	Tapetum PCD initiated
ST.6	Bicellular pollen stage	Tapetum degradation

^a^ Developmental events of wild-type anthers.

PCD: programmed cell death.

To investigate EAT1 expression during early meiosis, an *EAT1*pro-*EAT1-GFP* transcriptional fusion construct (Fig 1B) was introduced into male-sterile *eat1-4* plants homozygous for a putative null allele with a *Tos17*-retrotransposon insertion (S2A–S2F Fig). The transgenic plants recovered male fertility (Fig 1C), indicating that the EAT1-GFP protein is functional *in planta*. EAT1-GFP expression was bimodal at ST.2 and ST.5, as was mRNA expression, and the two expression peaks were clearly separated by the silent ST.4 (Fig 1D). Transcription of *AP25*, an aspartic protease gene required for tapetal PCD initiation [30], was fully dependent on EAT1 at ST.5 (S3 Fig, S1 Data), while no *AP25* transcript was detected at ST.2 or ST.3. These results confirm that the role of meiotic EAT1 is distinct from its postmeiotic role in tapetal PCD and further suggest that the EAT1 bHLH TF has distinct bHLH partners at these two developmental stages.

### Delayed and asynchronous male meiosis in the *eat1-4* mutant

**Fig 2:**
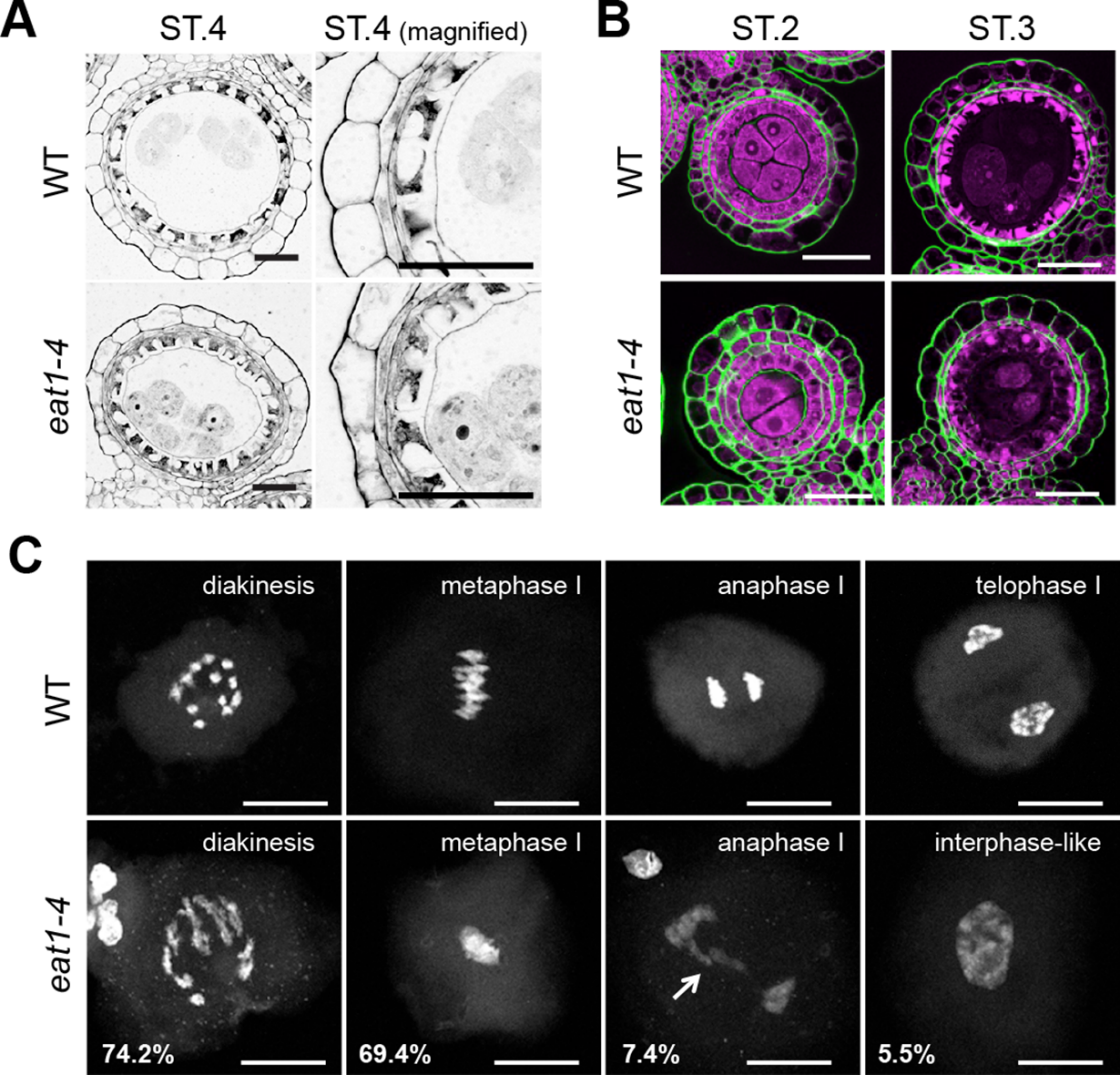
The *eat1-4* mutation affects meiotic chromosome condensation non-cell-autonomously. **(*A*)** Cross sections of anthers at late meiosis (ST.4). Tapetum and PMC formation of the *eat1-4* mutant was almost comparable to that of the wild-type (WT). Bars, 20 μm. **(*B*)** Accumulation (ST.2) and degeneration (ST.3) of β-1,4 glucan (green) at tapetal-cell and PMC walls. Nuclei were counterstained with propidium iodide (magenta). Bars, 20 μm. **(*C*)** Typical PMCs observed in respective meiotic stages in wild-type (top) and *eat1-4* anthers (bottom). Meiotic chromosomes, stained with 4',6-diamidino-2-phenylindole (DAPI), were decondensed frequently in the *eat1-4* PMCs. An arrow indicates lagging chromosomes. Bars, 20 μm.

In wild-type anthers, three concentric layers of somatic-wall cells at ST.1 become four layered at ST.2, and PMCs undergo meiosis at ST.3 and ST.4 (Fig 2A and S4A–S4C Fig). During ST.3-ST.4, the middle layer disappears, and during ST.5-ST.6, the tapetal layer degenerates by PCD (S4D and S4E Fig).

The *eat1-4* mutant phenotype was remarkable in postmeiotic ST.5 and ST.6 anthers, in which tapetal cells were unusually degenerated at ST.5, concurrent with abortive microspores and male sterility (S4I and S4J Fig). On the other hand, no morphological phenotype was found in earlier stages, ST.1 to ST.4 by light microscopy (Fig 2A and S4F–S4H Fig). Degradation of beta-1,4-glucan on cell walls of tapetal cells and PMCs occurred normally in *eat1-4* anthers at ST.2-ST.3 stages (Fig 2B). These observations were largely consistent with previous results [30].

We detected an unreported defect in male meiosis of *eat1-4* mutants: PMCs harbor aberrantly decondensed bivalent chromosomes frequently, 74.2% at diakinesis (n = 70) and 69.4% at metaphase I (n = 36) (Fig 2C). In addition, two out of 27 *eat1-4* PMCs at anaphase I harbored lagging chromosomes or chromosomal bridges, which were not found in the wild-type (n = 42) (Fig 2C). Another 5.5% *eat1-4* PMCs exhibited interphase-like nuclei with fully decondensed chromosomes (n = 163), in contrast to wild-type PMCs (n = 192, Fig 2C). In addition, meiotic division timing was retarded in mutant anthers, with asynchronous progression within an anther lobe (S5 Fig, S1 Data). Despite these meiotic defects, male meiosis could complete, but resulting microspores were aborted most likely by the aberrant tapetum, which normally secretes nutrients and exine components required during post-meiotic pollen development (S4J Fig). These results suggest that non-cell-autonomous signaling or some nutrient delivery between somatic tapetal cells and PMCs is mediated by EAT1 during meiosis, in addition to post-meiosis.

### EAT1 activates transcription of 101 loci encoding 24-*PHAS* RNAs

**Fig 3:**
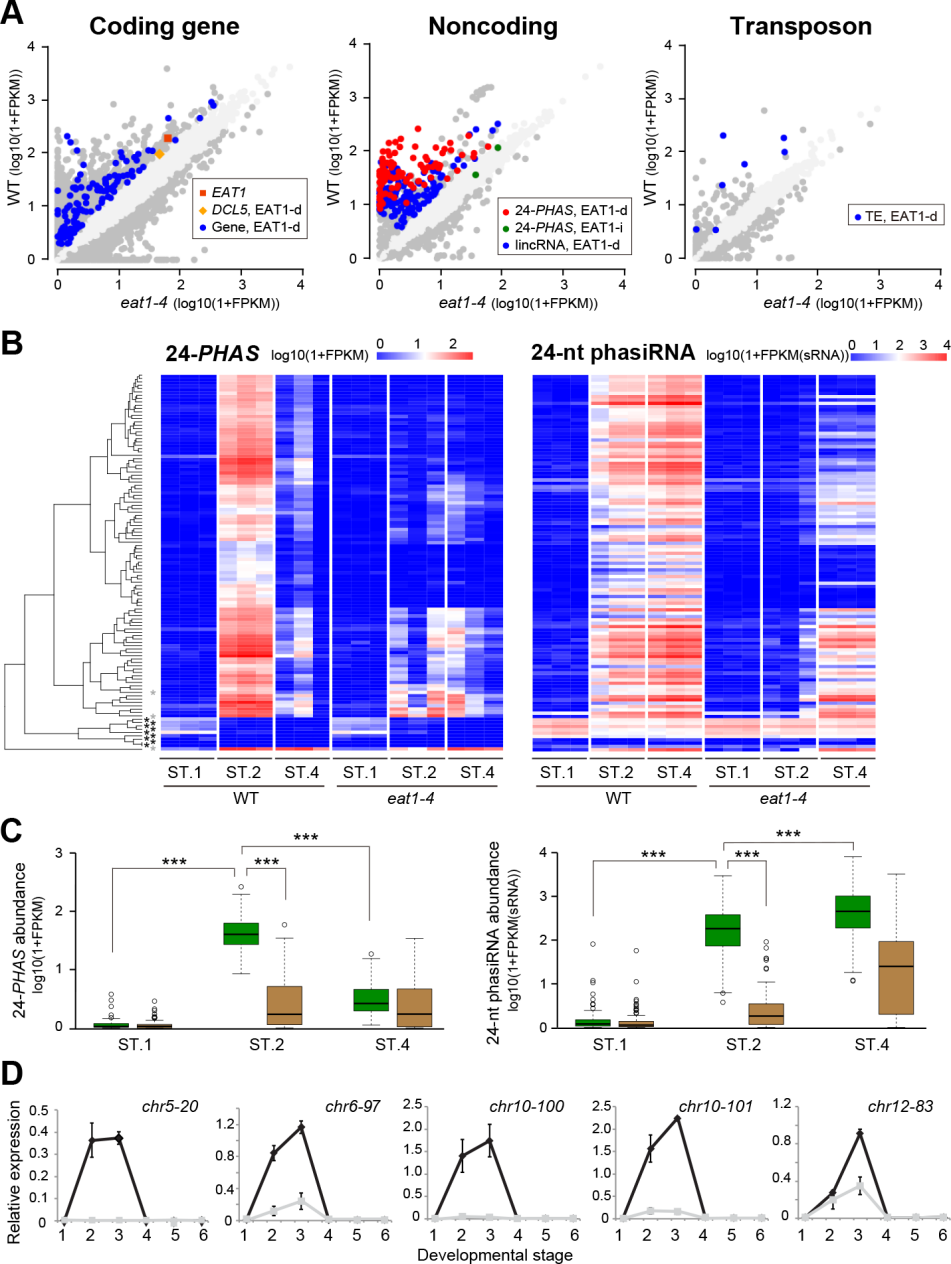
Identification and characterization of EAT1-dependent and early meiosis-enriched expression of 24-*PHAS* precursor transcripts. **(*A*)** Scatter plots of FPKM values for transcripts of 38,311 coding genes, 6,097 noncoding genes and 15,723 TE-like sequences, compared between the wild-type and *eat1-4* ST.2 anthers. EAT1-d and EAT1-i indicate that the transcripts show EAT1-dependent and EAT1-independent expressions, respectively. *DCL5* had slightly larger standard deviation of FPKM values in wild-type ST.2 (mandarin in left plot). In all plots, dark and faint gray spots represent transcripts whose FPKM values were ≥2-fold different between wild-type and *eat1-4* anthers, respectively. (***B***) Heatmaps representing the expression level of 113 loci encoding 24-*PHAS* precursor transcrips (left), and of 24-nt siRNAs (right) derived from the corresponding 24-*PHAS* loci (left). Each experiment includes three biological replicates. The leftmost dendrogram indicates the result of clustering of 24-*PHAS* expression patterns by R package, gplots. Asterisks indicate that the loci were silent through ST.1 to ST.4 stages (black) or showed EAT1-independent expression (grey). **(*C*)** Box plots representing 24-*PHAS* RNA density per locus (left) and 24-nt phasiRNA density per locus (right) in ST.1, ST.2 and ST.4 anthers of wild-type (green boxes) and *eat1-4* (brown boxes). *** indicate that difference is significant at *P* = 0.001 in Student's t-test. **(*D*)** qRT-PCR results of five 24-*PHAS* transcripts (*chr5-20*, *chr6-97*, *chr10-100*, *chr10-101* and *chr12-83*) in wild-type (black lines) and *eat1-4* anthers (gray lines). The bottom numbers correspond to anther developmental stages in Table 1. Relative expression values and standard errors were calculated by using three biological replicates.

To identify genes under the control of meiotically expressed EAT1, we conducted mRNA-seq experiments using whole anther samples and compared the data between wild-type and *eat1-4* plants. The data were obtained from three different meiotic stages: premeiosis (ST.1), early meiosis (ST.2) and late meiosis (ST.4), each with three biological replicates. 142,048,793 reads from wild-type and 146,928,874 reads from *eat1-4* anthers (S1 Table) in total were mapped to the rice genome. Of all 38,311 rice genes, 115 genes were defined to exhibit EAT1-dependent expression, which showed >2-fold greater Fragment per Kilobase per Million (FPKM) values in ST.2 anthers compared to *eat1-4* ST.2 anthers, and also compared to ST.1 and ST.4 anthers (Fig 3A, S2 Table). The ontology terms for 7 of 115 genes were enriched in lipid metabolism based on the agriGO algorithm [33] (S3 Table), implying that they function in pollen coat formation [34].

mRNA-seq also identified 6,097 regions generating long intergenic noncoding RNAs (lincRNAs), and 248 showed ST.2-enriched and EAT1-dependent expression (Fig 3A, S4 Table). Next, we conducted small RNA-seq (sRNA-seq) to ask whether these lincRNAs are small RNA precursors or not. 52,726,712 reads of total small RNAs extracted from wild-type and 62,364,061 from *eat1-4* anthers were mapped onto the rice genome (S1 Table). As a result, the 93 lincRNAs were defined as 24-*PHAS* RNAs, because a large number of 24-nt small RNAs were mapped in a 24-nt phasing manner on the lincRNA loci (see below for details). Of 44 24-*PHAS* loci previously reported [9, 10], 24 were included in the loci identified in this study. Another 8 loci, which were left out of our first selection by their length or overlapping coding genes, generated EAT1-dependent and ST.2-enriched 24-nt phasiRNAs (S4 Table), while the remaining 12 loci did not. Thus, adding the 8 loci, a total of 101 loci were specified as ST.2-enriched and EAT1-dependent 24-*PHAS* loci and analyzed hereafter.

Median FPKM values of 24-*PHAS* transcripts detected at the 101 loci in wild-type ST.2 anthers were 688-fold and 24-fold higher than those in ST.1 and ST.4 anthers, respectively. In addition, the values were 55-fold higher than in *eat1-4* anthers at ST.2 (Fig 3B and 3C, S4 Table). This result reconfirmed the EAT1-dependent and early meiosis-enriched nature of 24-*PHAS* transcripts. This trend was reproducible in qRT-PCR of five 24-*PHAS*s (Fig 3D, S1 Data). In contrast, most 24-nt RNAs from the corresponding *PHAS* loci were abundant not only in ST.2, but also in ST.4 anthers (Fig 3B and 3C, S4 Table), implying slower turnover of small RNAs than precursor transcripts.

**Fig 4:**
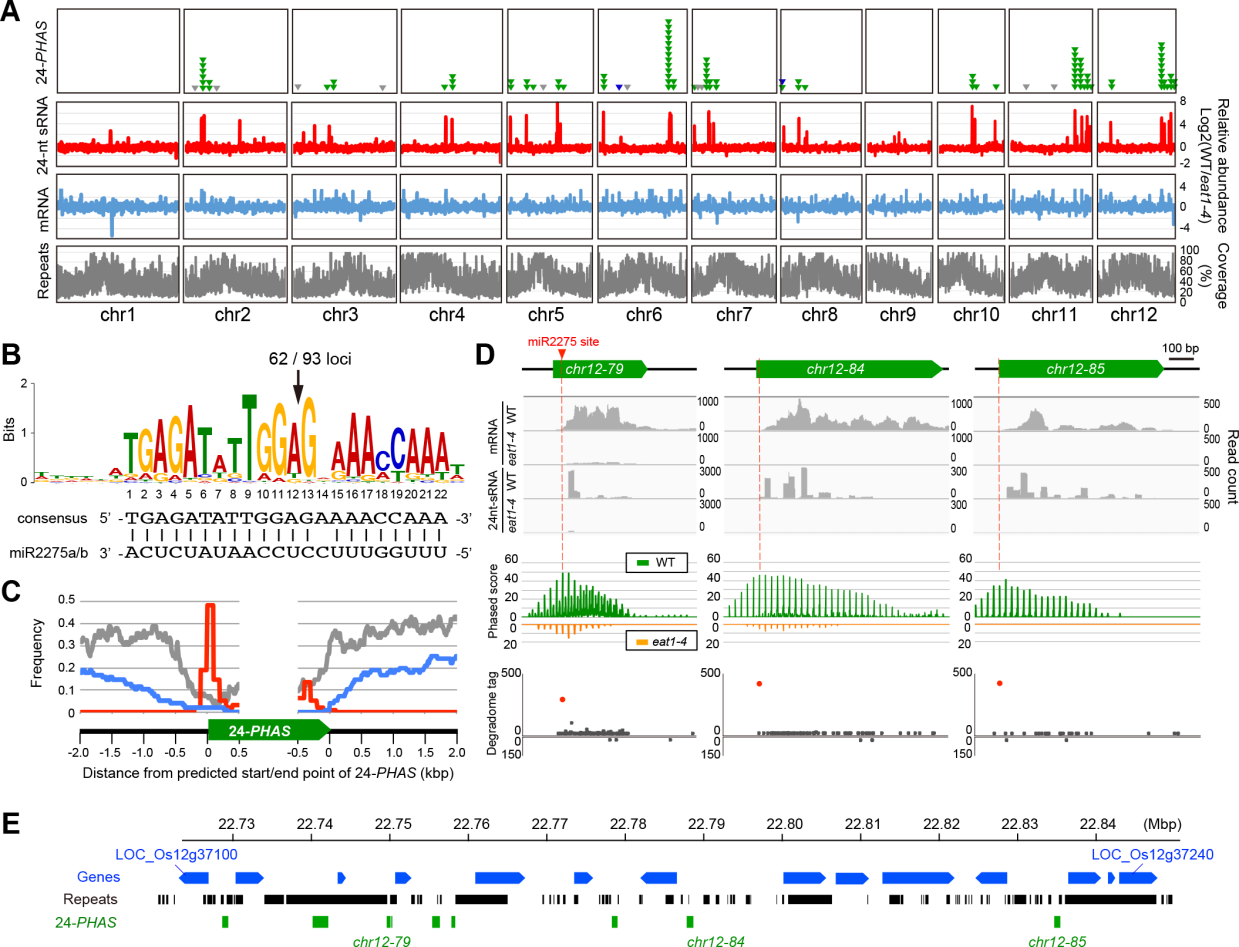
Characterization of 24-*PHAS* loci on rice genome. (***A***) A genome-wide distribution of 24-*PHAS* loci. From top to bottom, the numbers of 24-*PHAS* loci (101 green triangles correspond to 24-*PHAS* loci showing an EAT1-dependent and ST.2-enriched expression, 9 gray triangles are previously reported 24-*PHAS* loci silent through ST.1 to ST.4 and 3 blue triangles are those showing EAT1-independent expression), the amounts of 24-nt sRNA-seq (red), mRNA-seq reads (blue) rated by subtraction of *eat1-4* values from wild-type values (see Methods), and frequencies of repetitive sequences including TEs (gray charts). The horizontal length of each box corresponds to the physical distance of respective rice chromosomes. (***B***) A conserved sequence logo found in upstream of ninety-three 24-*PHAS* loci detected by MEME program [35], which are potentially targeted by miR2275. The arrow indicates the predicted cleaved position by DCL1 and miR2275 complex [10]. (***C***) Frequency of repetitive sequence (grey), gene coding region (blue) and miR2275 targeted site (red) around 24*-PHAS* loci. The data was examined in 93 24-*PHAS* transcripts with conserved miR2275 targeted sites. The reason why a small peak of miR2275 target site appeared at the 3’ end of 24-*PHAS* is that some 24-*PHAS* loci were relatively small in length (~ 500 bp). (***D***) Characterization of three 24-*PHAS* loci. From the top to the bottom, the graphs indicate the mapping results of mRNA-seq and 24-nt sRNA-seq reads (gray histograms), the 24-nt phasing pattern (green and orange charts), and the plot of read counts from the degradome-seq using young panicles of *indica* variety, 93–11 [38]. The degradome analysis revealed that the cleavage of three 24-*PHAS* transcripts frequently occurs at the position shown in (***B***), within the predicted miR2275 sites (red dots), while few degradome-seq reads were mapped onto both sense and antisense strands of other regions (gray dots). Reads were depicted by IGV [78]. (***E***) An example of distribution of EAT1-dependent 24-*PHAS*-loci cluster (green boxes) on the long arm of chromosome 12, with the context of surrounding genes (blue) and repetitive sequences (black).

The 101 *PHAS* loci were unevenly distributed in the genome as reported previously [9], except for chromosomes 1 and 9, and many loci formed several clusters on each chromosome (Fig 4A, S4 Table). Sequence comparison by the MEME program [35] demonstrated that 93 out of 101 24-*PHAS* loci conserved 22-mer sequence complementary to mature miR2275 (Fig 4B and 4C, S4 Table). The miR2275 sites were conserved at the 5'-region in 92 loci (Fig 4C, S4 Table), consistent with previous results that 22-mer miRNA triggers one-hit processing [36, 37]. The phased pattern tended to start at the 13th position in the 22-mer miR2275 site in most of 24-*PHAS* loci (Fig 4D). This position corresponded to the cleavage site of the AGO1/miR2275 complex reported previously [10]. Consistent with this, the degradome data from the *indica* rice variety [38] demonstrated that the cleavage actually occurred at the same position relative to the miR2275 complementarity in 62 of 93 24-*PHAS* loci (Fig 4B and 4D, S4 Table), and that almost of lincRNAs detected here were the unprocessed, primary 24-*PHAS* RNAs.

Of 24-nt small RNAs mapped on 93 24-*PHAS* loci, the 77.1% reads from wild-type ST.2 and ST.4 anthers showed a 24-nt phased pattern which starts from putative AGO1/miR2275 cleavage site (S6 Fig, S1 Data), indicating that 24-nt small RNAs produced from these loci were processed by DCL5.

Most 24-*PHAS* loci were mapped to unique or low copy regions (Fig 4C and 4E, S1 Data). Only 7 of the so-far reported 15,723 TEs showed ST.2-enriched and EAT1-dependent expression (Fig 3A right, S2 Table). We concluded that meiotic 24-nt phasiRNAs originate from 101 intergenic 24-*PHAS* loci and that they have a role distinct from TE silencing.

### EAT1 binds 24-*PHAS* and *DCL5* promoters in meiotic tapetum

**Fig 5:**
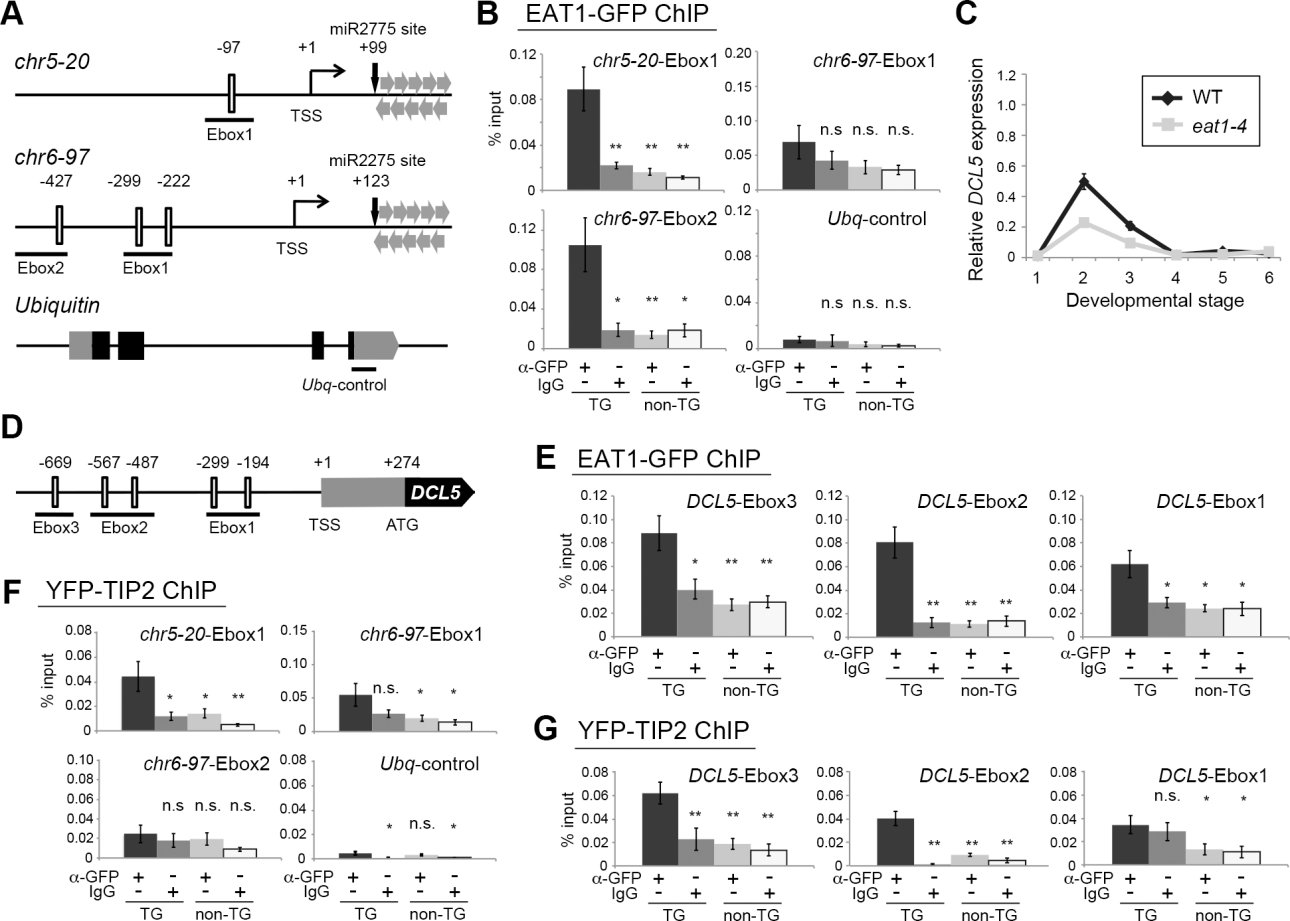
EAT1 and TIP2 bind E-box motifs upstream of 24-*PHAS* loci and *DCL5* gene. **(*A*)** Schematic illustrations of genomic compositions of the 5ʹ upstream regions of two 24-*PHAS* loci, *chr5-20* and *chr6-97*, in addition to the coding region of the *Ubiquitin* gene as a negative control. Open boxes indicate the position of consensus E-box motifs. The number at the top of each motif shows a distance (bp) from the transcription start site (TSS). Regions underlined were used in the ChIP-qPCR assay. Grey and closed boxes in the *Ubiquitin* represent untranslated and coding regions, respectively. (***B***) ChIP-qPCR results of 24-*PHAS* promoters using transgenic (TG) plants expressing EAT1-GFP. IgG and non-TG plants were used as negative controls. n.s.; not significant. * and **; significant at *P* = 0.05 and *P* = 0.01 in Student's t-test, respectively, less than the leftmost positive ChIP result in each graph. **(*C*)** qRT-PCR results of *DCL5* mRNA in wild-type and *eat1-4* anthers. Relative expression values and standard errors were calculated by three biological replicates. The bottom numbers correspond to anther developmental stages in Table 1. **(*D*)** Genomic composition of the 5ʹ upstream region of the *DCL5* gene. **(*E*)** ChIP-qPCR results of *DCL5* promoters using TG plants expressing EAT1-GFP. **(*F*)** ChIP-qPCR results of 24-*PHAS* promoters using TG plants expressing YFP-TIP2. **(*G*)** ChIP-qPCR results of *DCL5* promoters using TG plants expressing YFP-TIP2. In ChIP-qPCR analyses, relative abundance and standard errors were calculated by two or three biological replicates each subjected to three PCR replications.

Chromatin-immunoprecipitation (ChIP)-qPCR analysis was performed to examine EAT1-binding to the upstream *cis* sequences of two 24-*PHAS* loci (*chr5-20* and *chr6-97*) using EAT1-GFP-expressing plants. Both sequences included E-box motifs, short CANNTG sequences potentially targeted by bHLH proteins [39] (Fig 5A). The *chr5-20*-Ebox1 was enriched 5.4-fold and the *chr6-97*-Ebox2 was enriched 6.1-fold in ChIP of EAT1-GFP-expressing anthers (Fig 5B, S1 Data), suggesting that EAT1 has a potential to target 24-*PHAS* loci.

The above results prompted the idea that EAT1 activates genes including 24-nt phasiRNA biogenesis-related (24-PBR) genes. Indeed, *DCL5* was 2.1-fold downregulated in *eat1-4* ST.2 anthers in mRNA-seq analysis (Fig 3A, S2 Table, S1 Data), and this reduction was confirmed by qRT-PCR (Fig 5C, S1 Data). ChIP using EAT1-GFP-expressing anthers and anti-GFP antibody displayed enrichment of the Ebox2 and Ebox3 upstream of *DCL5* by 6.5- and 2.7-fold, respectively (Fig 5D and 5E, S1 Data). In contrast, no EAT1 binding was detectable in two other *DCL* family genes, *DCL3a*, responsible for long miRNA production required for cytosine DNA methylation and TE-associating 24-nt siRNA synthesis [40, 41], and *DCL4*, involved in 21-nt phasiRNA production [10] (S7A and S7B Fig, S1 Data), despite the presence of E-box motifs. A substantial abundance of *DCL5* transcripts still in *eat1-4* anthers (Fig 5C) implies a possibility that other TFs participate in this process.

The expression of 24-PBR genes other than *DCL5* was examined. DCL1 and RDR6 are respectively required for processing of miR2275 precursors and RNA double-strand formation [10, 21]. *DCL1* and *RDR6* transcripts were abundant in ST.2 anthers; however, both were also abundant in ST.1 and ST.4 anthers and were unaffected by the *eat1-4* mutation (S8A Fig, S1 Data), indicating that expression of *DCL1* and *RDR6* is EAT1 independent and not restricted to meiotic stages. Transcripts of *pri-miR2275a*/*b*, the precursors of mature miR2275, were enriched in ST.2 anthers. In contrast to 24-*PHAS*s and *DCL5*, the amount of *pri-miR2275* transcripts was elevated in the *eat1-4* mutant (S8A Fig, S1 Data). *pri-miR2275b* promoter sequences were not enriched in ChIP of EAT1-GFP-expressing anthers, despite containing E-box motifs (S8C and S8D Fig, S1 Data).

**Fig 6:**
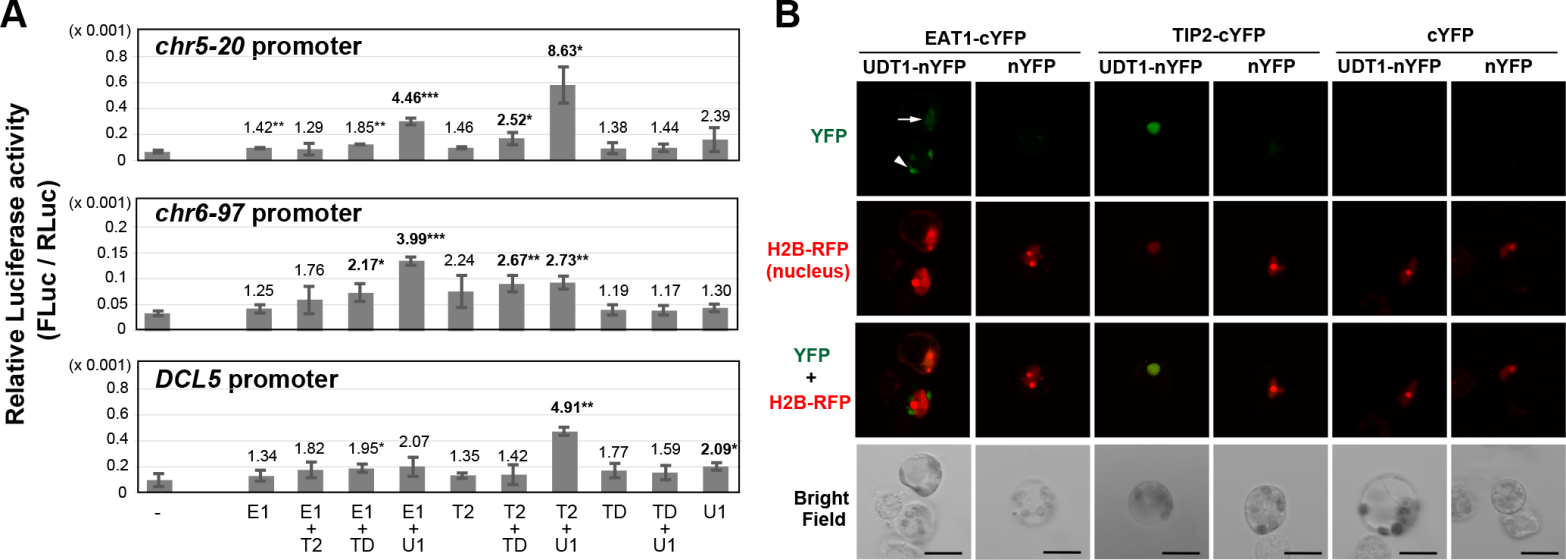
EAT1 and TIP2 activate the promoter activity of 24-*PHAS* loci and the *DCL5* gene in interaction with UDT1. **(*A*)** The results of the transient expression assay. Any one or two effector plasmids encoding EAT1 (E1), TIP2 (T2), UDT1 (U1) and TDR (TD) proteins were cotransfected with the reporter constructs into rice protoplasts. The reporter carries a 2-kbp promoter region of the 24-*PHAS*s (*chr5-20*, *chr6-97*) or *DCL5*, fused with the firefly *Luciferase*. The configuration of all constructs were shown in S10A Fig. The number above each bar is the fold change of the Luciferase activity compared to the negative control without the promoter (leftmost bars). *, ** and ***; the significant fold changes at *P* = 0.05, 0.01 and 0.001 in Student’s t-test, respectively, compared to the negative control. Error bars indicated standard deviation of three biological replicates. The significant >2 fold changes were in bold. **(*B*)** The BiFC results of EAT1-UDT1 an TIP2-UDT1 cotransfections.

To investigate the EAT1 ability to promote the transcription of 24-*PHAS* and *DCL5* loci, we performed the transient expression assay. The bHLH proteins have homo- and heterodimerization ability [42]. Thus, the effector construct encoding any two of EAT1, TIP2, UDT1 and TDR was cotransfected with the 24-*PHAS* or *DCL5* promoter (p*PHAS*, p*DCL5*)-*Luciferase* fusion reporter into rice protoplasts (S10A Fig), and the promoter activity was measured. The activity of two p*PHAS*s was significantly 4.46 (*chr5-20*) and 3.99-fold (*chr6-97*) elevated in EAT1-UDT1 cotransfection, compared to the no effector control (Fig 6A). However, contrary to expectations, the same combination displayed insignificant effects on the p*DCL5* (Fig 6A). Little effect on p*PHAS*s nor p*DCL*5 was observed in the transfection of EAT1 alone and EAT1-TIP2, while the EAT1-TDR cotransfection slightly affected the activity of p*PHAS*s (1.85 and 2.17 fold) and p*DCL5* (1.95 fold) (Fig 6A). Interestingly, EAT1-UDT1 cotransfection induced the p*EAT1* activity by greater 7.61 fold (S10B Fig), while it was slightly upregulated by the EAT1-TDR cotransfection (1.58 fold). Cotransfection of EAT1 with TIP2, TDR or UDT1 displayed no significant effect on the p*DCL3a* (S10B Fig).

To examine the protein-protein interaction between EAT1 and UDT1, we performed the bimolecular fluorescence complementation analysis (BiFC) in rice protoplasts. EAT1 fused with the C-terminal split of YFP (EAT1-cYFP and cYFP-EAT1) gave positive BiFC signals when coexpressed with UDT1-nYFP (Fig 6B, S11A and S11B Fig), while they tended to be detectable faintly in the nucleus (Fig 6B, S11A Fig arrows) or intensely in the cytoplasm (Fig 6B, S11A Fig arrowhead). In both cases, the positive signals were always more intense compared to negative controls (Fig 6B, S11A–S11C Fig).

The above results demonstrate that the meiotic EAT1 TF promotes the transcription of 24-*PHAS* precursors and the *EAT1* gene itself by interacting with UDT1 at the molecular level. EAT1 also promotes the *DCL5* transcription, but likely with an unknown bHLH partner.

### TIP2 also activates 24-*PHAS* and 24-PBR gene transcription

Next, we examined the protein function of TIP2, an EAT1 paralog [27, 28, 37]. The *tip2-2* loss-of-function allele newly identified in this study had a T-DNA insertion in the third intron (S2G–S2L Fig, S1 Data). In transverse sections of developing anthers (S4K–S4X Fig), the wild-type tapetal and middle layer cells have dense cytoplasm (S4M–S4N Fig), however, in the mutants the cell layers had sparse cytoplasm at ST.3 and ST.4 (S4T–S4U Fig). The central PMCs were eventually collapsed probably due to malformed somatic layers (S4V Fig). These results reconfirmed the previous proposal that TIP2 is essential for differentiation of precursor cells into middle layer and tapetal cells [27, 28].

When a *TIP2*pro-*YFP-TIP2* transcriptional fusion construct was introduced in the *tip2-2* mutant, YFP-TIP2 signals were intensified in tapetal cell nuclei at ST.2 and ST.3, and in addition, weaker signals were observed in the nuclei of middle layer cells (S9A–S9C Fig). TIP2 protein expression was EAT1 independent, while in contrast, EAT1 expression was TIP2 dependent in transgenic plants (S9D and S9E Fig, S1 Data).

qRT-PCR indicated that the levels of 24-*PHAS*, *DCL5*, and *pri-miR2275a/b* transcripts at ST.2 were severely reduced in *tip2-2* anthers (S8B Fig, S1 Data). Using YFP-TIP2-expressing plants, the region upstream of the 24-*PHAS* locus (*chr5-20*-Ebox1) was 4.3-fold enriched in ChIP of YFP-TIP2 (Fig 5F, S1 Data), and the upstream Ebox2 and Ebox3 sequences of *DCL5* also showed 8.1 and 3.4-fold enrichment, respectively (Fig 5G, S1 Data).

In the transient expression assay, TIP2-UDT1 cotransfection resulted in a significant increase of the p*PHAS* (8.63 fold on *chr5-20* and 2.73 fold on *chr6-97*) and p*DCL5* activities (4.91 fold) (Fig 6A). TIP2-TDR cotransfection also elevated the p*PHAS* activity (2.52 and 2.67 fold) (Fig 6A). Both TIP2-UDT1 and TIP2-TDR activated the p*EAT1* by 5.72 and 2.35 fold, respectively (S10B Fig), consistent to TIP2-dependent EAT1-GFP expression in transgenic plants (S9E and S9F Fig) and to the previous results [27, 28]. The BiFC assay clearly indicated that TIP2 has a potential to interact with UDT1 (Fig 6B and S11A–S11C Fig).

Collectively, these results suggest that TIP2 has the potential to activate transcription of both 24-*PHAS*s and *DCL5* by interacting with UDT1 at the molecular level in early meiosis.

### A subset of 24-nt phasiRNAs is bound by the Argonaute expressed in male meiocytes

**Fig 7:**
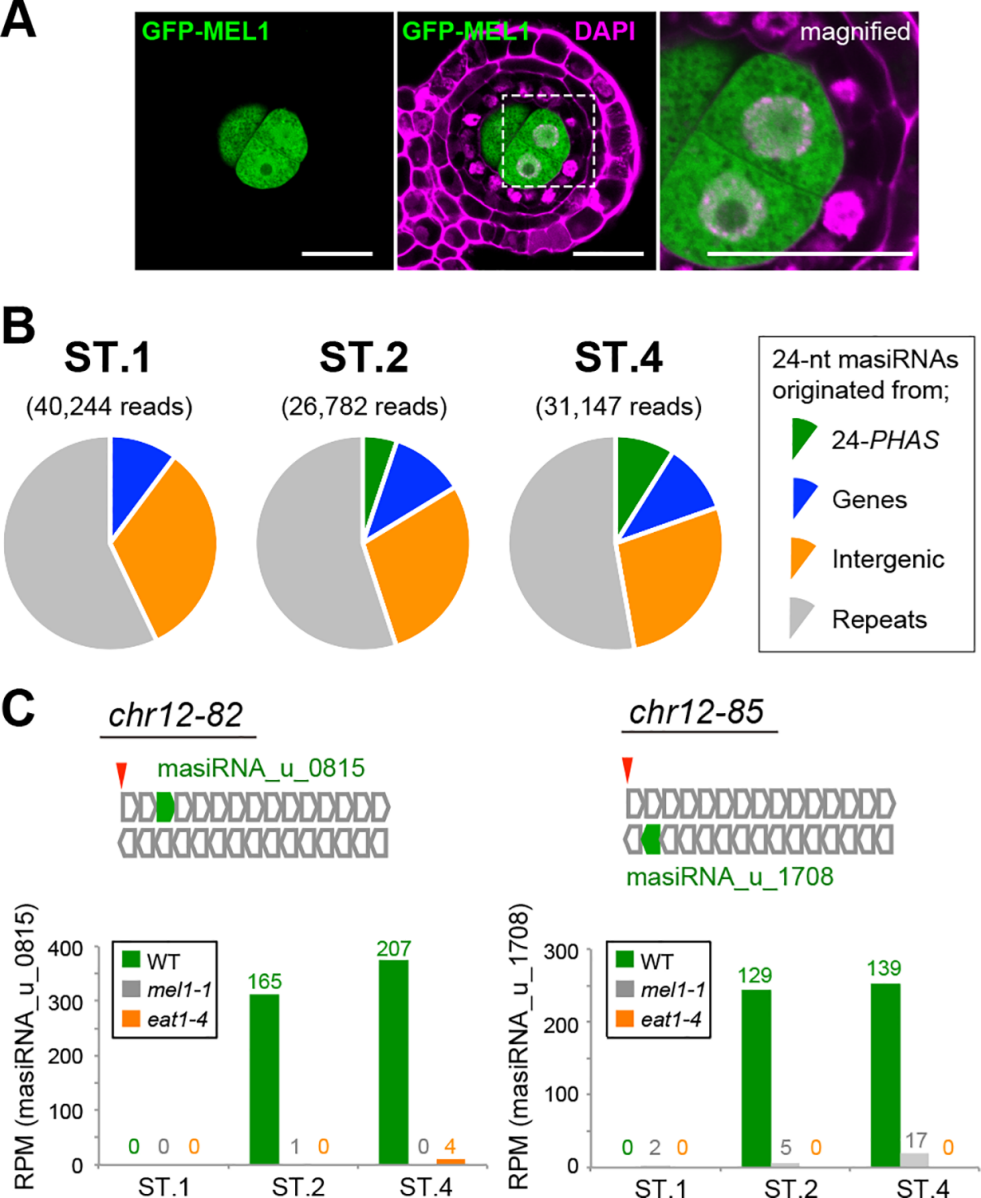
The MEL1 Argonaute protein associates with EAT1-dependent 24-nt phasiRNAs in male meiocytes. **(*A*)** MEL1-GFP was specifically expressed in the male germline in ST.2 anthers in *MEL1*pro-*MEL1*-*GFP* transgenic plants. Bars, 10 μm. **(*B*)** Pie-charts representing the ratios of 24-nt MEL1-associating siRNAs (masiRNAs) originated from 24-*PHAS* loci, protein-coding genes, intergenic regions except for 24-*PHAS* loci and repetitive regions, in wild-type samples through ST.1, ST.2 and ST.4 stages. The numbers with parentheses indicate the read counts of 24-nt masiRNAs extracted from MEL1-IPseq results. **(*C*)** The mapping mode of 24-nt masiRNAs on two 24-*PHAS* loci, for example. Tandem arrays of open box-arrows (top) represent the 24-nt phased interval pattern on both strands of each *PHAS* locus. Green box-arrows are 24-nt masiRNAs exactly fitting to the interval. Red arrowheads indicate conserved miR2275 targeted sites. Each bar graph (bottom) indicates RPM values of the 24-nt masiRNA (masiRNA_u_0815 or _1708) in wild-type (WT), *mel1-1* and *eat1-4* anthers. The numbers at the top of bars represent a total read counts of 24-nt masiRNAs with two biological replicates.

Small RNAs are sorted to confer association with specific Argonaute family proteins [43]. MEL1 is a rice Argonaute protein whose function is well characterized in meiosis, and is abundantly expressed in male and female meiocytes, but not in surrounding somatic cells [7]. As supporting this result, the MEL1-GFP expression was limited to premeiotic and meiotic PMCs in transgenic plants (Fig 7A). Here we used MEL1 Argonaute as an indicator for the 24-nt phasiRNA existence or absence in male meiocytes, and performed RNA-immunoprecipitation sequencing using anti-MEL1 antibody (MEL1-RIPseq) in flowers at three stages; ST.1, ST.2 and ST.4.

1,711,113, 1,361,031 and 2,679,034 reads of 24-nt small RNAs from three stages were obtained from MEL1-RIPseq of wild-type, *eat1-4* and *mel1-1* flowers, respectively (S1 Table). After subtraction of *mel1-1* mutant results and mapping onto the rice genome, 2,110 species (98,145 reads) were defined as canonical 24-nt MEL1-associating siRNAs (masiRNAs) (S1 Data). Through all three stages, 24-nt masiRNAs originated from repetitive sequences (57.1, 55.0 and 52.7% at ST.1, ST.2 and ST.4, respectively), intergenic regions other than 24-*PHAS* loci (32.6, 28.7 and 27.6%) and protein coding regions (10.2, 11.1 and 10.7%) (Fig 7B). In contrast, 24-nt masiRNAs from 24-*PHAS* loci were detected in ST.2 and ST.4 (5.2 and 9.0%), but hardly detected in ST.1 anthers (<0.1%) (Fig 7B). This result corresponds to the temporal expression pattern of EAT1-dependent 24-nt phasiRNAs (Fig 3B right). In *eat1-4* mutant, masiRNAs from 24-*PHAS* loci occupied few portion of masiRNA reads even in ST.2 (< 0.1%) and ST.4 (<0.5%) in addition to ST.1 (<0.1%) (S12A Fig, S1 Data). MEL1 preferentially bound 24-*PHAS*-derived 24-nt masiRNAs with a 5'-terminal cytosine (S12B Fig), consistent with the 5'-end preference of MEL1 [11]. The *mel1* mutant anthers displayed only a few 24-nt RNA reads in MEL1-RIPseq in each stage (S5 Table, S1 Data).

The mapping mode of 24-nt masiRNAs was shown in two 24-*PHAS* loci for example (*chr12-82* and *chr12-85*, Fig 7C). On the *chr12-82* locus, 165 and 207 reads of only a 24-nt masiRNA species (masiRNA_u_0815) were mapped at the third phase of the sense strand in ST.2 and ST.4 anthers, respectively (Fig 7C left). A significant reduction of the masiRNA_u_0815 in male-sterile *eat1-4* plants (Fig 7C, S5 Table) confirmed their origin in anthers, not in pistils, although MEL1 is expressed in both male and female cells [7]. A similar tendency was found in the *chr12-85* and masiRNA_u_1708 (Fig 7C right).

Collectively, above results indicate that a subset of EAT1-dependent 24-nt phasiRNAs, at least the versions retaining 5'-terminal cytosine, was bound by MEL1.

## Discussion

### EAT1 and TIP2 triggers meiotic 24-nt phasiRNA biogenesis

Previous studies unveil the complicated interaction of four bHLH proteins, UDT1, TIP2, EAT1 and TDR, in aliphatic metabolism and PCD in tapetal cells for rice pollen development. In post-meiosis, the TIP2/TDR heterodimer directly activates the *EAT1* transcription, and the EAT1 competes for the TIP2/TDR activity [28], because EAT1 also dimerizes with TDR [30]. EAT1 activates transcription of *AP25* and *AP37*, both required for tapetal PCD [30].

This study gave new insights in the relationship of tapetal bHLH proteins during early meiosis. First, the EAT1 expression is bimodal, not only in post-meiosis, but also in early meiosis (Fig 1). Second, the transient expression assay suggests a possibility that the transcription of *EAT1* gene during early meiosis is activated by the TIP2/UDT1 heterodimer, and reinforced by the EAT1/UDT1 (Fig 6, S10 Fig). Third, both EAT1 and TIP2 can activate transcription of 24-*PHAS* lincRNAs and the *DCL5* gene in tapetum during early meiosis (Figs 3, 5 and 6A, S10B Fig). The activation by EAT1 is thought to be independent of that by TIP2, because of no interaction between two proteins as previously reported [27, 28] and shown in this study (Fig 6A, S10 Fig). In these two pathways, UDT1 is a strong candidate for the dimerization partner of EAT1 and TIP2 (Fig 6B, S11 Fig), while dimerization of unknown bHLH proteins with EAT1 is supposed in the *DCL5* transcription (Fig 6A). In the *udt1* mutant, the tapetum is aberrantly vacuolated and the tetrads are degenerated during meiosis [32]. This observation is consistent to the idea that UDT1 acts with TIP2 and EAT1 in 24-nt phasiRNA biogenesis in rice anther tapetum during meiosis. The temporal replacement of binding partners from UDT1 to TDR may enable EAT1 and TIP2 to switch downstream targets from meiotic phasiRNA production to postmeiotic tapetal PCD induction.

In this study, we performed mRNA-seq and sRNA-seq to estimate 24-nt phasiRNA production only in the *eat1-4* (Fig 3), but not in the *tip2-2*. This is because in the *tip2* mutant, tapetum is replaced by undifferentiated cell layers [27, 28] (S4U–S4X Fig), and the absence of 24-*PHAS* and *DCL5* transcripts is possibly a by-product of the missing tapetum. However, the results that at least two 24-*PHAS* transcripts enriched at ST.2 were transcribed EAT1-independently (green spots in Figs 3A and 5C), and that non-negligible amounts of 24-*PHAS* and *DCL5* transcripts are expressed still in *eat1-4* anthers at ST.2 (Fig 3B left, Fig 5C). Taken together with the results of ChIP-qPCR and transient expression assay, it is obvious that TIP2 has an indispensable role in 24-nt phasiRNA production.

The maize (Zm) bHLH122, the EAT1 ortholog, also shows bimodal expression [25], and MALE STERILE23 (MS23), the TIP2 ortholog, promotes the expression of *bHLH122*/*ZmEAT1*, *DCL5*, 24-*PHAS* transcripts and meiotic 24-nt phasiRNAs [12, 25]. A positive interaction in the yeast two hybrid analysis (Y2H) is reported between MS32/ZmUDT1 and bHLH122/ZmEAT1, consistent to the results of this study (Fig 6, S10 and S11 Figs). Thus, the bHLH TF-mediated mechanism underlying specification and development of tapetum is well conserved in rice and maize, and commonly coupled with meiotic small RNA production. A contradiction between maize and rice is in the relationship of TIP2 and UDT1. In maize, a negative Y2H interaction of MS23/ZmTIP2 and MS32/ZmUDT1 is reported [25], whereas rice TIP2 and UDT1 interact with each other at the molecular level (Fig 6B) and promote the activity of p*PHAS*s, p*DCL5* and p*EAT1* (Fig 6A, S10B Fig). Further analyses will be necessary for conservation and differentiation of tapetal bHLH protein functions in these monocot model plants.

### A possibility of intercellular mobilization of EAT1-dependent phasiRNAs in anthers

The observation that a subset of tapetum-originating phasiRNAs was sorted to MEL1 Argonaute, which is abundantly expressed in PMCs but not in tapetal cells (Fig 7). Though the possibility that 24-nt phasiRNA functions mainly in tapetum cannot be excluded, the result of this study suggests another possibility that the 24-nt phasiRNA is mobile between somatic and reproductive cells in rice anthers. This idea is attractive and proposed previously [12, 13], but should be considered carefully. It is difficult to exclude the possibility that 24-nt phasiRNAs are produced cell-autonomously in PMCs by EAT1 and/or TIP2-independent pathways, for example, DNA double-strand break (DSB)-induced small RNAs [44, 45]. However, we think this unlikely, because *mel1* mutant anthers with few meiotic DSBs in male meiocytes [46] produce a robust level of 24-nt phasiRNAs (S1 and S4 Tables). In addition, few amounts of 24-nt phasiRNAs are detectable in *eat1-4* anthers (Fig 3C, S3 Table). A recent study unveiled that 24-nt phasiRNA and miR2275 expression is depleted in two rice mutants, *multiple sporocytes1* (*msp1*) and *tpd1-like gene in rice1a* (*tdl1a*), in which a subset of inner anther-wall cells turn into PMCs [24, 47]. In maize, the *ms23* anther lacking the tapetum fails to produce 24-nt phasiRNAs, but the *ocl4* anther developing the tapetum succeeds [12]. These results suggest that 24-nt phasiRNA production occurs exclusively in tapetum, consistent to the conclusion of this study.

An alternative possibility is that precursor *PHAS* transcripts or their processed intermediates are transferred from tapetum, and processed into mature 24-nt phasiRNAs by 24-PBR components in PMCs. TIP2 and EAT1 are detectable in somatic companions, but hardly in PMCs (Fig 2D, S9C Fig), implicating that most of DCL5-mediated 24-*PHAS* processing is completed in anther tapetum. However, to answer the above question, further analyses for tissue-specific expression of precursor transcripts and 24-PBR components are required.

Another question for the intercellular small-RNA movement is whether the undetectable level of MEL1 proteins accumulates in tapetal cells during meiosis and associates with tapetum-expressing 24-nt phasiRNAs. However, *MEL1* mRNA expression is ranked at the top 1.7 percentile (the 629th highest) of all protein-coding transcripts expressed in ST.2 anthers (S13 Fig), and as reflecting the higher mRNA level, the MEL1-GFP signal in male meiocytes made a striking contrast to undetectable signals in somatic anther cells in transgenic plants (Fig 7A). Thus, small RNAs immunoprecipitated with somatic MEL1 are, if any, hard to be detected in the RIPseq analysis of anther samples, that is, the MEL1 RIPseq data of this study largely comes from the masiRNA population derived from male meiocytes. In any case, rigorous verification requires some breakthrough technologies for live-imaging of small RNAs or sequestering them into the particular cell type, such as tapetal cells.

Molecular transport in plants occur either symplastically through plasmodesmata, or apoplastically across the cell membrane, cell walls and intercellular space [48]. Tapetal cells and PMCs are connected with plasmodesmata and form symplastic continuity by the onset of meiotic leptotene (ST.2 in this study) [49, 50], when EAT1-dependent meiotic 24-nt phasiRNAs are produced in tapetal cells (Fig 3). This interconnection is broken by callose accumulation [49]. Callose is the highly impermeable polysaccharide distinct from cellulose [50], and can be a barrier for apoplastic molecular movement. However, in ST.2 anthers, cellulosic components still remain between tapetum and PMCs (Fig 2B), in turn, callose accumulation is absent or less. Thus, both symplastic and apoplastic movements are currently possible mechanisms underlying meiotic phasiRNA movement in anthers.

Taking previous findings into consideration, we propose that considerable amounts of 24-nt meiotic phasiRNAs are imported from tapetum to PMCs during early meiosis in rice. If it is true, not only the phasiRNAs with the 5'-teminal cytosine (C-terminal phasiRNAs), but also non-C-terminal ones are supposed to move together in the intercellular movement, because the enrichment of C-terminal phasiRNAs in MEL1-RIPseq in this study is simply due to the selectivity of MEL1 [11]. The analysis of other Argonautes expressed in PMCs will be beneficial to trace tapetum-originating non-C-terminal phasiRNAs.

### Implication of 24-nt phasiRNA function in meiotic chromosome remodeling

Functions of other Argonaute proteins in plant meiosis still remain to be debated. Rice flowers highly express many Argonaute proteins in addition to MEL1/AGO5 (AGO1b, AGO1d, AGO2b, AGO4a, AGO9 and AGO18) [8, 51], whose meiotic roles are largely unknown. *Arabidopsis* AGO4 plays important roles in chromosome condensation and segregation during the first meiotic division [52], comparable to rice EAT1 function in male meiosis (Fig 2C). ZmAGO104, orthologous to *Arabidopsis* AGO9, is also required for meiotic chromosome condensation [53]. In either case, the relationship of Argonaute/small RNA complexes to the nuclear RdDM and histone modification will be one of the most important questions regarding epigenetic regulation of plant meiosis.

Dukowic-Schulze et al. [13] unveiled that both 21- and 24-*PHAS* precursor loci showed higher DNA methylation in all cytosine contexts (CG, CHG, CHH, where H represents A, T or C) in isolated maize PMCs. The highest context was CHH methylation, implying that reproductive phasiRNAs are involved in RNA-directed DNA methylation (RdDM) in PMCs. RdDM includes both *de novo* DNA methylation and histone H3 lysine-9 (H3K9) methylation in plants [54–59]. Supporting this idea, MEL1 is thought to govern meiosis-specific chromatin remodeling accompanying dynamic alteration in H3K9 dimethylation [46].

Meiosis is a special type of cell division to transmit new haplotypes to the next generation, and additionally, to survey incompatibilities in ploidy levels and chromosomal structures between both parents. This process must be strictly regulated by complicated mechanisms genetically and epigenetically. Recent genome-wide studies have revealed that small RNA-mediated and non-cell-autonomous regulation is likely general in reproduction of eukaryotic species. Further analyses of tapetum-expressing bHLH TFs and meiotic phasiRNAs in anthers will bring new epigenetic insights into plant reproduction systems.

## Materials and methods

### Plant materials

The *eat1-4* mutant is a *Tos17* insertion line produced from the rice variety, cv. Nipponbare [60], NF9876, kindly provided by the Rice Genome Resource Center, Japan. The *mel1-1* mutant [7], another *Tos17* line with the Nipponbare background, was kindly provided by the National Bioresource Project (NBRP) Rice, conducted by the Japan Agency for Medical Research and Development (AMED). The *tip2-2* mutant is a T-DNA tag line with the genetic background of cv. Dongjin [61, 62], 1B-24309, kindly provided by Dr. G. An (POSTECH, Korea). All plants were grown in moist chambers, greenhouses, and/or open paddy fields at the National Institute of Genetics (NIG), Mishima, Japan. Plant genotypes were determined by PCR using GoTaq Green Master Mix (Promega) and gene-specific and T-DNA/*Tos17-*internal primers (S6 Table).

### Histology

Rice spikelets were fixed in PMEG buffer (50 mM PIPES, 10 mM EGTA, 5 mM MgSO_4_, and 4% glycerol, pH 6.8) containing 4% paraformaldehyde (PFA) for 3 h and washed six times in PMEG buffer for 2 hours. After dehydration using ethanol series, they were embedded in Technovit7100 resin (Heraeus Kulzer), sectioned in 2 μm thick slices using a LM2255 microtome (Leica Microsystems), stained with 0.1% toluidine blue O (Wako Pure Chemicals) and photographed using a BX50 light microscope (Olympus) and a DP50 camera system (Olympus). Cellulosic cell wall staining was conducted according to the method described previously [63]. Fluorescent images were captured using a Fluoview FV300 CLSM system (Olympus), and pseudo-colored and merged using Photoshop CS4 (Adobe Systems Inc.).

### Construction and transformation of fluorescent-tagged proteins

*EAT1*pro-*EAT1*-*GFP* (Fig 1D) was constructed as follows. The 5.3 kbp *Hin*dIII-*Xho*I genomic fragment including the upper half of the *EAT1* gene and its promoter region was subcloned from a BAC clone, OSJNBa0010K21, into the pBluescriptII (pBSII)-SK(-) vector. The 1.2 kbp *Xho*I-*Eco*RV fragment including the 3’ downstrem region of the *EAT1* gene was also subcloned into another pBSII-SK(-), and from this plasmid, the 2.0 kbp *Xho*I-*Eco*RV fragment harboring a *sGFP* sequence just in the front of the *EAT1* stop codon was constructed using *EAT1*-specific primers, bHLH141stop-BamHI/bHLH141XhoI-BamHI and bHLH141stop-NotI/M13-Rev, and a CaMV35S-sGFP(S65T)-nos3ʹ vector [64], kindly provided by Dr. Y. Niwa (Shizuoka U., Japan). The resultant 5.3 kbp and 2.0 kbp fragments were inserted into a pPZP2H-lac binary vector [65] to assemble *EAT1*pro-*EAT1*-*GFP*.

*TIP2*pro-*YFP*-*TIP2* (S8A Fig) was constructed as follows. The 6.6 kbp genomic fragment, including the entire *TIP2* gene with 4 kbp upstream and 0.5 kbp downstream sequences, was cut out from a rice BAC clone OSJNBa0001E17 by digestion with *Spe*I, and inserted into pBSII-SK(-) vector. From the 6.6 kbp fragment, the 1.8 kbp *Hin*dIII-*Sal*I fragment including the translational initiation site (TIS) was subcloned into pBSII-SK(-). From this plasmid, the *YFP* sequence was inserted just in front of TIS by using *TIP2*-specific primers bHLH142start-NcoI/M13-Rev and bHLH142start-BsrGI/T7-EcoRI and a pEYFP vector (a cloning vector with *EYFP* sequence in pUC18 backbone). Then, the 1.8 kbp fragment with the *YFP* sequence was inserted back into the original 6.6 kbp genomic fragment/pBSII-SK(-) plasmid. The resultant 7.4-kbp insert was digested, blunt-ended, and reinserted into pGWB601 binary vector [66], kindly provided by Dr. T. Nakagawa (Shimane U., Japan). In case of *TIP2*pro-*TIP2*-*YFP* (S8D Fig), the *TIP2* stop codon in the above 6.6 kbp genomic fragment/pBSII-SK(-) was replaced by *YFP* sequence by using *TIP2*-specific primers, bHLH142stop-NcoI/M13-20 and bHLH142stop-BsrGI/M13-Rev, and a pEYFP. Finally, the 7.4 kbp of *TIP2*pro-*TIP2*-*YFP* insert was assembled in the pPZP2H-lac. In above constructions, KOD-FX DNA polymerase (TOYOBO) was used for PCR.

In *MEL1*pro-GFP-*MEL1* construction, the *GFP* sequence was inserted just in front of *MEL1* TIS in pKN16, a binary vector containing the 18 kbp *MEL1* genomic fragment [7]. Two DNA fragments, corresponding to 5ʹ upstream and 3ʹ downstream regions of *MEL1* TIS, were amplified from pKN16 with primer pairs up_nf/up_nr and up_atgf/up_r, respectively. Linker-attached *sGFP* coding sequence was amplified from CaMV35S-sGFP(S65T)-nos3ʹ with ngfp_f/ngfp_r primers. The PCRs were conducted using a PrimeSTAR Max DNA polymerase (TaKaRa). The three amplified DNA fragments were mixed with the *Nru*I-*Asc*I-digested pKN16 and incubated with an In-Fusion HD enzyme premix (TaKaRa) to assemble *MEL1*pro-GFP-*MEL1*, following manufacturer’s instructions. All the primer sequences for the construction were listed in S6 Table.

The constructs were transformed into rice calli using agrobacterium-mediated transformation [67], in which Hygromycin B (50 mg/L in media; Wako Pure Chemicals) or glufosinate-ammonium PESTANAL (5 mg/L in media; Sigma-Aldrich) was used for a positive selection.

### Observation of GFP and YFP signals in rice anthers

Anthers embedded in 6% SeaKem GTG agarose (Lonza) were sliced into 50 μm thickness by MicroSlicer DTK-ZERO1 (D.S.K.), and mounted on slide grasses with VECTASHIELD (Vector Laboratories) containing DAPI. Fluorescent images were captured using Fluoview FV300 CLSM system (Olympus).

### Meiotic chromosome observation

Spikelet (lemma) and anther lengths were measured under SMZ645 stereo microscopy (Nikon). 0.8–1.2 mm anthers were fixed with 4% PFA/PMEG and provided for chromosome observations as previously described [7]. Fluorescent images of DAPI were taken as described above.

### RNA extraction and quantitative RT-PCR (qRT-PCR)

Anther or spikelet samples were separated by their lengths as corresponding to ST.1-ST.6 stages (S7 Table), immediately frozen with liquid nitrogen in microtubes, and stored at -80°C until use. Total RNAs were extracted from the samples using TRIzol reagent as manufacturer’s recommendation (Life Technologies), and treated with DNase I (TaKaRa). In qRT-PCR, 1 μg of total RNA was reverse-transcribed by oligo(dT)_12-18_ primer (Life Technologies) and SuperscriptIII reverse transcriptase (Life Technologies). The products were 20-fold diluted and supplied for real-time qPCR using gene-specific primers (S6 Table), KAPA SYBR FAST universal qPCR Kit (KAPA Biosystems) and Thermal Cycler Dice Real Time System (TaKaRa). Rice *Ubiquitine* gene was used as an internal standard.

### mRNA-seq, sRNA-seq and data analyses

Total RNAs were extracted from ST.1, ST.2 and ST.4 anthers of wild-type and *eat1-4* plants, three biological replicates each. For mRNA-seq, 1 μg of total RNA was subjected to library construction using KAPA stranded mRNA-seq Kit Illumina Platforms (KAPA biosystems). Eighteen libraries differentially indexed by FastGene Adapter kit (Nippon Genetics) were multiplexed (9 per lane) and sequenced by HiSeq2500 (Illumina) with SR50 (single ended). Adapter sequences were removed *in silico* using R package QuasR [68].

mRNA-seq reads were mapped on the rice genome IRGSP1.0 using Tophat (v2.0.14) [69]. Differential expression analysis of annotated genes were conducted using Cuffdiff2 program [70]. The genes fulfilling all of the following conditions were regarded as EAT1-dependent and ST.2-enriched genes; (1) genes showing >2-fold higher FPKM values in wild-type ST.2 anthers than the values in wild-type ST.1 and ST.4 anthers, (2) genes showing >2-fold higher FPKM values in wild-type ST.2 anthers than the values in *eat1-4* ST.2 anthers, and (3) genes with each standard deviation less than a half of the FPKM mean value of three replicates in wild-type ST.2 anthers. The lincRNAs were determined by Cuffdiff2 (merged.gtf), in which protein-coding genes were removed as referring to MSU7.0 annotation, and unannotated but transcribed genomic regions larger than 200 bp were extracted. FPKM values of lincRNAs were calculated by BEDtools [71]. Furthermore, EAT1-dependent and ST.2-enriched lincRNAs were extracted according to the same conditions described above for coding genes.

For sRNA-seq, 1 μg of total RNA was provided for library construction by NEBNext Multiplex Small RNA Library Prep Set for Illumina (New England BioLabs). The libraries were 9-plexed per lane and sequenced by HiSeq2500 (illumina) with SR52, a 2-bp extended version of SR50, for higher-quality sequencing. After trimming by QuasR, 24-nt long sRNA-seq reads were extracted by ShortRead [72], and mapped to the rice IRGSP1.0 genome using Tophat, in which reads having >50 multi-hits on rice genome or any mismatches were cut off (-N 0 -g 50). If 24-nt RNAs with >10 FPKM values were mapped on each of EAT1-dependent and ST.2-enriched lincRNA loci identified above, the loci were defined as 24-*PHAS* loci. Regional abundance of mRNA-seq and 24-nt sRNA-seq reads mapped on the rice genome (shown in Fig 4A) was calculated in a sliding window (window; 50 kbp, step; 25 kbp) by BEDtools. Conserved motifs were searched in each 24-*PHAS* locus, in addition to 200 bp regions both upstream and downstream sequences, by MEME SUITE program [35]. Phased scores were calculated as described by Howell et al. [73].

### Degradome-seq data analysis

A degradome-seq dataset from young panicles of *indica* rice variety, cv. 93–11, was obtained from Sequence Read Archive of DNA Data Bank of Japan (DDBJ-SRA) under the accession code SRR034102 [38]. Adaptor sequence and low-quality reads were removed using FASTX-toolkit (http://hannonlab.cshl.edu/fastx_toolkit/) and the reads retaining 20- or 21-nt length were mapped onto rice IRGSP1.0 genome using Bowtie 2 [74]. The frequency of 5’-end of mapped reads were manually examined within and around 24-*PHAS* loci identified in this study (S4 Table).

### 5ʹ RACE

To determine the TSS of two 24-*PHAS*s and a *pri-miR2275* (*chr5-20*, *chr6-97* and *pri-miR2275b*), the standard 5ʹ rapid amplification cDNA end (5ʹ RACE) method was applied using a GeneRacer kit (Thermo Fisher Scientific), total RNA from ST.2 wild-type anther, and gene specific primers (S6 Table). Eight clones from each locus were sequenced using a BigDye Terminator v3.1 cycle sequencing kit (Applied Biosystems) and a PRISM 3130xl sequencer (Applied Biosystems) and the end of the longest read(s) was marked as TSS.

### Chromatin immunoprecipitation (ChIP)-qPCR

Rice young panicles from transgenic derivatives were fixed, and the anthers at early meiosis (around 0.5 mm) were supplied for ChIP as described previously [75]. The anti-GFP antibody No.598 and the normal rabbit IgG (both from MBL International) were used for positive and negative ChIP experiments, respectively. The extracted DNAs were analyzed by real-time qPCR using region-specific primers (S6 Table). The 1/10 volume of chromatin-containing samples without IP treatment was prepared for the input samples.

### Transient expression assay in rice protoplast

The 2-kbp upstream sequences from the translational start site of *24-PHAS* (*chr5-20*, *chr6-97*), *DCL5*, *EAT1* and *DCL3a* genes, all originated from the *japonica* rice cv. Nipponbare, were inserted in the upstream of the firefly *Luciferase* CDS and the nopaline synthase (nos) terminator. This reporter construct was cloned into pBSII-SK(-) plasmid (S10A Fig). For the effector construct, the cauliflower mosaic virus 35S (CaMV35S) promoter was fused with the cDNAs of *EAT1*, *TIP2*, *TDR* and *UDT1* genes, originated from Nipponbare ST.2 anthers, with the nos terminator. and cloned into pBSII-SK(-) (S10A Fig). For normalization of the firefly Luciferase activity, the *Luciferase* cDNA of *Renilla reniformis* were fused with the CaMV35S promoter and the nos terminator, inserted into pBSII-SK(-) (S10A Fig), and cotransfected with the effector and reporter constructs as an internal control in all experiments. All PCR primers for the above constructions were listed in S6 Table. PrimeSTAR Max DNA polymerase (TaKaRa) was used for PCR amplification according to the manufacturer's instruction. Protoplast preparation from rice seedlings, transfection of plasmids, and protein extraction from protoplasts were according to the method previously described [76]. The Luciferase activity was detected using Dual-Luciferase Reporter Assay System (Promega) and Filter MAX F5 multi-mode microplate reader (Molecular Devices).

### Bimolecular fluorescence complementation (BiFC) of bHLHs

A pair of split YFP vectors (pBS-35S-nYFP and pBS-35S-cYFP) were kindly provided by Drs. D. Tsugama (Hokkaido U., Japan) and T. Takano (The U. of Tokyo, Japan) [77]. Each of *EAT1*, *TIP2* and *UDT1* cDNAs, originated from Nipponbare ST.2 anthers, was inserted into the either of upstream or downstream of both pBS-35S-nYFP and pBS-35S-cYFP vectors. For the nuclear marker, the rice *Histone 2B* (*H2B*) cDNA were fused with the maize *Ubiquitine* promoter and in-frame with the *tagRFP* gene (Evrogen), cloned into pPZP2H-lac binary vector [65], and cotransfected with a pair of split YFP constructs in all experiments. All PCR primers used here were listed in S6 Table. PrimeSTAR Max DNA polymerase (TaKaRa) was used for PCR. The protoplast preparation and plasmid transfection were same with the method described above. Fluorescent images were captured by Fluoview FV300 CLSM system (Olympus) and processed by Photoshop CS4 (Adobe systems Inc.), under the identical conditions and parameters through all experiments. We tried all sixteen combinations of split YFP constructs to assess EAT1-UDT1 and TIP2-UDT1 interactions, and thirteen combinations of negative controls. However, UDT1-cYFP and cYFP-UDT1 gave the intense signal in a single transfection as negative controls, and excluded from the assay. Then, the total eight combinations of EAT1-UDT1 (EAT1-cYFP/UDT1-nYFP, EAT1-cYFP/nYFP-UDT1, cYFP-EAT1/UDT1-nYFP, cYFP-EAT1/nYFP-UDT1) and TIP2-UDT1 (TIP2-cYFP/UDT1-nYFP, TIP2-cYFP/nYFP-UDT1, cYFP-TIP2/UDT1-nYFP, cYFP-TIP2/nYFP-UDT1) were assayed.

### RNA immunoprecipitation (RIP)-seq and analysis of masiRNAs

RIP fractions from wild-type, *mel1-1* and *eat1-4* flowers at ST.1, ST.2 and ST.4, each of which included two biological replicates, were obtained using anti-MEL1 antibody as described previously [11]. Library construction, sequencing, adapter trimming, size filtration and mapping to rice genome were done as well as sRNA-seq methods described above. Reads per million (RPM) values were calculated in the respective 24-nt RNA sequences and compared among wild-type, *mel1-1* and *eat1-4* fractions. In this process, 24-nt masiRNAs were defined in 24-nt RNA sequences as having ≥15 RPM detected in wild-type ST.1, ST.2 or ST.4 flowers, and ≥RPM 4-fold enriched in wild-type compared to *mel1-1*.

### Gene accession numbers

*EAT1*; Os04g0599300, *TIP2*; Os01g0293100, *TDR*; Os02g0120500, *UDT1*; Os07g0549600, *MEL1*, Os03g0800200, *DCL5*; Os10g0485600, *DCL3a*; Os01g0909200, *DCL4*; Os04g0509300, *DCL1*; Os03g0121800, *RDR6*; Os01g0527600, *AP25*; Os03g0186900. (Rice Annotation Project Database (RAP-DB) (http://rapdb.dna.affrc.go.jp)).

## Supporting information

S1 Fig Expression of *bHLH* genes during anther development. Expression patterns of four tapetum-related *bHLH* genes; *UDT1*, *TDR*, *TIP2* and *EAT*1, in wild-type (cv. Nipponbare) anther development. The bottom numbers of the developmental stage correspond to Table 1. Relative expression values and standard errors were calculated usng three biological replicates.
(TIF)

S2 Fig Male sterile phenotype of *eat1-4* and *tip2-2* mutants. **(*A*** and ***G*)** Genomic structure of *EAT1* and *Tos17* insertion of *eat1-4* (***A***) and *TIP2* and T-DNA insertion of *tip2-2* (***G***).
**(*B*** and ***H*)** qRT-PCR results of underlined regions of *EAT1* transcript in wild-type and *eat1-4* flowers (***B***) and of *TIP2* transcript in wild-type and *tip2-2* flowers (***H***). In (***B***) and (***H***), total RNAs from early meiotic flowers (around 2.0 mm) long were used.
**(*C*** and ***I*)** Flower morphology of *eat1-4* (***C***) and *tip2-2* (***I***). Bars, 1 mm.
**(*D*, *E*, *J*** and ***K*)** I_2_KI staining of mature pollen in the anther of *EAT1* wild type **(*D*)**, *eat1-4* mutant **(*E*)**, *TIP2* wild type **(*J*)**, and *tip2-2* mutant **(*K*)**. Bars, 100 μm.
**(*F*** and ***L*)** *EAT1* mRNA expression during anther development in wild type (cv. Nipponbare) and *eat1-4* plants (***F***) and *TIP2* mRNA expression in wild type (cv. Dongjin) and *tip2-2* anthers (***L***). In (***F***), expression data of *EAT1* transcripts in wild-type anthers were identical to those of S1 Fig.
In qRT-PCR analyses, relative expression values and standard errors were calculated by three biological replicates.
(TIF)

S3 Fig Expression pattern of *AP25* function in tapetal PCD. *AP25* expression during anther development in *eat1-4* (left), *tip2-2* (right) and their respective wild-type siblings. Relative expression values and standard errors were calculated by three biological replicates.
(TIF)

S4 Fig Anther morphology of *tip2-2* and *eat1-4* mutants. **(*A*** to ***J*)** Anther cross-sections of wild-type (*EAT1*) **(*A*** to ***E*)** and *eat1-4* **(*F*** to ***J*)**. The cross-sections of ST.4 and their magnified views are shown in Fig 2A.
**(*K*** to ***X*)** Anther cross-sections of wild-type (*TIP2*) **(*K*** to ***Q*)** and *tip2-2* (***R*** to **X**).
**(*A***, ***F***, ***K***, and ***R*)**: ST.1; **(*B***, ***G***, ***L*** and ***S*)**: ST.2; **(*C***, ***H***, ***M***, and ***T*)**: ST.3; **(*N*** and ***U*)**: ST.4; **(*D***, ***I***, ***O*** and ***V*)**: ST.5; **(*E***, ***J***, ***P*** and ***W*)**: ST.6; **(*Q*** and ***X*)**: magnified view of ST.4.
Bars, 20 μm.
(TIF)

S5 Fig Retarded and asynchronous male meiosis in *eat1-4* anthers. (***A***) Box plots of spikelet lengths in each meiosis I stage in wild-type and *eat1-4* mutant anthers. Zyg.; early zygotene, Pac.; pachytene, Dip.; diplotene, Div; the stage including diakinesis, metaphase I, anaphase I, dyad, and second division. n.s. and *** indicate no significance and significance at *P* = 0.001 (Student's t-test), respectively, between the wild-type and *eat1-4*. Arrowheads indicate average values.
(***B***) Column charts showing the spectrum of meiotic stages in single wild-type and *eat1-4* anthers.
(TIF)

S6 Fig Box plots of 24-nt small RNA reads exhibiting 24-nt phased pattern on 93 24-*PHAS* loci. Of 254,163 and 877,203 reads of 24-nt small RNAs from ST.2 and ST. 4 anthers, 329,112 (ST.2) and 1,138,234 reads (ST.4) were defined as in-phase reads for the 24-nt phased interval that starts from the predicted miR2275 cleavage site (Fig 4B and 4C) on 93 24-*PHAS* loci identified in this study. Then, the frequency of in-phase reads to total reads were box-plotted. The sRNA-seq reads from three replicates were combined in each stage and plotted. The median values were 0.814 and 0.820 in wild-type ST.2 and ST.4 samples, respectively.
(TIF)

S7 Fig *DCL3a* and *DCL4* were not targeted by EAT1 and TIP2. **(*A*)** Structure of 5ʹ upstream regions of *DCL3a* and *DCL4*. The diagrams are equivalent to Fig 5D.
**(*B*** and ***C*)** ChIP-qPCR results of *DCL3a* and *DCL4* promoter region using transgenic (TG) plants expressing EAT1-GFP **(*B*)** and YFP-TIP2 **(*C*)**. n.s.; not significant. * and **; significant at *P* = 0.05 and *P* = 0.01 in Student's t-test, respectively, less than the leftmost positive ChIP result in each graph. Relative abundance and standard errors were calculated by two or three biological replicates each subjected to three PCR replications.
**(*D*)** qRT-PCR results of *DCL3a* and *DCL4* during anther development of *eat1-4*, *tip2-2* and their respective wild-type siblings. Relative expression values and standard errors were calculated by three biological replicates.
(TIF)

S8 Fig Expression of 24-nt phasiRNA biogenesis-related genes in *eat1-4* and *tip2-2* anthers. (***A***) qRT-PCR results of 24-nt phasiRNA biogenesis-related genes, *DCL1*, *RDR6*, and two *pri-miR2275* transcripts in wild-type and *eat1-4* anthers.
(***B***) qRT-PCR results of five 24-*PHAS* transcripts, *DCL5*, *DCL1*, *RDR6*, and two *pri-miR2275* transcripts in wild-type and *tip2-2* anthers. In qRT-PCR analyses, relative expression values and standard errors were calculated by three biological replicates.
(***C***) Schematic illustration of genomic compositions of the 5ʹ upstream regions of *pri-miR2275b* locus. The diagrams are equivalent to Fig 5A.
**(*D*** and ***E***) ChIP-qPCR results of *pri-miR2275b* promoters using TG plants expressing EAT1-GFP **(*E***) and YFP-TIP2 (***E***). In ChIP-qPCR analyses, relative abundance and standard errors were calculated by two or three biological replicates each subjected to three PCR replications. n.s.; not significant. * and **; significant at *P* = 0.05 and *P* = 0.01 in Student's t-test, respectively, less than the leftmost positive ChIP result in each graph.
(TIF)

S9 Fig TIP2 expression and localization in anther wall cells at early meiosis. **(*A*)** Diagram of the *TIP2*pro-*YFP-TIP2* transcriptional fusion construct. Closed and grey boxes indicate protein coding and untranslated regions, respectively.
**(*B*)** *tip2-2*/*tip2-2* flowers of T_0_ plants carrying *EAT1*pro-*EAT1-GFP* (#1, #2) and an empty vector. Bars, 1 mm.
**(*C*)** YFP-TIP2 signals (green) in developing anther sections from ST.1 to ST.5. in a transgenic plant harboring *TIP2*pro-*YFP-TIP2*. YFP-TIP2 signals were intensified in tapetal nuclei (arrowhead) and also detected in middle layer nuclei (arrow) in ST.2 and ST. 3 anthers, and not detected in the ST.2 anther from the negative control (n.c., right most panel). Bars, 20 μm.
(***D*** and ***E***) TIP2-YFP expression and localization in wild-type and *eat1-4* ST.2 anthers (***D***) and EAT1-GFP expression and localization in wild-type and *tip2-2* ST.2 anthers (***E***). Bars, 20 μm.
(***F*** and ***G***) Expression pattern of *TIP2* mRNA in wild-type and *eat1-4* anthers (***F***), and *EAT1* mRNA in wild-type and *tip2-2* anthers (***G***). Relative expression values and standard errors were calculated by three biological replicates.
(TIF)

S10 Fig The configuration of reporter and effector constructs for the transient expression assay, and results of the assay for *EAT1* and *DCL3a* promoters. **(*A*)** Schematic diagrams of the reporter, effector and internal control constructs used in the transient expression assay. The reporter carries a 2-kbp promoter region of the 24-*PHAS*s (*chr5-20*, *chr6-97*), *DCL5*, *EAT1* or *DCL3a* fused with the firefly *Luciferase*. CaMV35S; cauliflower mosaic virus 35S promoter, nos; nopaline synthase terminator.
**(*B*)** The results of the transient expression assay. Any one or two effector plasmids encoding EAT1 (E1), TIP2 (T2), UDT1 (U1) and TDR (TD) proteins were cotransfected with the reporter constructs into rice protoplasts. The number above each bar is the fold change of the Luciferase activity compared to the negative control without the effector (leftmost bars). *, ** and ***; the significant fold changes at *P* = 0.05, 0.01 and 0.001 in Student’s t-test, respectively, compared to the negative control. Error bars indicated standard deviation of three biological replicates. The significant >2 fold changes were in bold.
(TIF)

S11 Fig Positive interaction between EAT1 and UDT1 and between TIP1 and UDT1 in BiFC. (A) BiFC results of EAT1-cYFP and TIP2-cYFP constructs combined with a nYFP-UDT1 sonstruct and those of negative control combinations. (B) BiFC results of cYFP-EAT1 and cYFP-TIP2 constructs combined with a UDT1-nYFP construct and those of negative control combinations. (C) BiFC results of cYFP-EAT1 and cYFP-TIP2 constructs combined with a nYFP-UDT1 construct. Some negative control results common in Fig 6B and S11 Fig. were indicated by empty boxes.
(TIF)

S12 Fig MEL1 RIP-seq for *eat1-4* mutant anthers and the sequence logo of MEL1-associating 24-nt small RNAs. (A) Pie-charts representing the ratios of 24-nt MEL1-associating siRNAs (masiRNAs) originated from 24-*PHAS* loci, protein-coding genes, intergenic regions except for 24-*PHAS* loci and repetitive regions, in *eat1-4* anthers at ST.1, ST.2 and ST.4 stages. The numbers with parentheses indicated the read counts of 24-nt masiRNAs extracted from MEL1-IPseq results.
(B) The sequence logos generated from 68 species of 24-nt masiRNAs mapped onto 24-*PHAS* loci (top), and from all 2,110 species of 24-nt masiRNAs mapped on the rice genome (bottom).
(TIF)

S13 Fig *MEL1* is highly expressed in rice meiotic anthers. A histogram representing the distribution of FPKM values of all 38,311 rice genes in wild-type ST.2 anthers. The area where *MEL1* included was indicated by an arrowhead.
(TIF)

S1 Data Data underlying Fig 3, Fig 4, Fig 5, Fig 6, S1 Fig, S2 Fig, S3 Fig, S5 Fig, S6 Fig, S7 Fig, S8 Fig, S9 Fig and S12 Fig. (XLSX)

S1 Table Number of reads in mRNA-seq, sRNA-seq and MEL1-RIPseq analyses. (XLSX)

S2 Table Summary of coding genes and transposons showing EAT1-dependent expression pattern. (XLSX)

S3 Table Results of GO enrichment analysis of 115 EAT1-dependent genes. (XLSX)

S4 Table Summary of EAT1-dependent 24-*PHAS*, EAT1-independent 24-*PHAS* and EAT1-dependent lincRNA (non-24-*PHAS*) loci. (XLSX)

S5 Table Summary of masiRNAs identified in the EAT1-dependent 24-*PHAS* loci. (XLSX)

S6 Table Primer sequences used in this study. (XLSX)

S7 Table Anther length and corresponding developmental stages in *eat1-4* and *tip2-2* plants. (DOCX)

S8 Table Identifiers of mRNAseq, sRNAseq and MEL1-RIPseq data deposited to DDBJ. (XLSX)

## References

[1] Kim VN, Han J, Siomi MC. Biogenesis of small RNAs in animals. Nat Rev Mol Cell Biol. 2009;10(2): 126–39. doi: 10.1038/nrm2632 19165215 10.1038/nrm2632

[2] Vaucheret H. Post-transcriptional small RNA pathways in plants: mechanisms and regulations. Genes Dev. 2006;20: 759–71. doi: 10.1101/gad.1410506 16600909 10.1101/gad.1410506

[3] Siomi MC, Sato K, Pezic D, Aravin AA. PIWI-interacting small RNAs: the vanguard of genome defence. Nat Rev Mol Cell Biol. 2011;12(4): 246–58. doi: 10.1038/nrm3089 21427766 10.1038/nrm3089

[4] Gou LT, Dai P, Yang JH, Xue Y, Hu YP, Zhou Y et al Pachytene piRNAs instruct massive mRNA elimination during late spermiogenesis. Cell Res. 2014;24(6): 680–700. doi: 10.1038/cr.2014.41 24787618 10.1038/cr.2014.41 PMC4042167

[5] Lee HC, Gu W, Shirayama M, Youngman E, Conte D Jr., Mello CC. *C*. *elegans* piRNAs mediate the genome-wide surveillance of germline transcripts. Cell. 2012;150(1): 78–87. doi: 10.1016/j.cell.2012.06.016 22738724 10.1016/j.cell.2012.06.016 PMC3410639

[6] Shirayama M, Seth M, Lee HC, Gu W, Ishidate T, Conte D Jr., et al piRNAs initiate an epigenetic memory of nonself RNA in the *C*. *elegans* germline. Cell. 2012;150(1): 65–77. doi: 10.1016/j.cell.2012.06.015 22738726 10.1016/j.cell.2012.06.015 PMC3597741

[7] Nonomura KI, Morohoshi A, Nakano M, Eiguchi M, Miyao A, Hirochika H, et al A germ cell specific gene of the *ARGONAUTE* family is essential for the progression of premeiotic mitosis and meiosis during sporogenesis in rice. Plant Cell. 2007;19(8): 2583–94. doi: 10.1105/tpc.107.053199 17675402 10.1105/tpc.107.053199 PMC2002623

[8] Kapoor M, Arora R, Lama T, Nijhawan A, Khurana JP, Tyagi AK, et al Genome-wide identification, organization and phylogenetic analysis of Dicer-like, Argonaute and RNA-dependent RNA Polymerase gene families and their expression analysis during reproductive development and stress in rice. BMC Genomics. 2008;9: 451 doi: 10.1186/1471-2164-9-451 18826656 10.1186/1471-2164-9-451 PMC2576257

[9] Johnson C, Kasprzewska A, Tennessen K, Fernandes J, Nan GL, Walbot V, et al Clusters and superclusters of phased small RNAs in the developing inflorescence of rice. Genome Res. 2009;19(8): 1429–40. doi: 10.1101/gr.089854.108 19584097 10.1101/gr.089854.108 PMC2720183

[10] Song X, Li P, Zhai J, Zhou M, Ma L, Liu B, et al Roles of DCL4 and DCL3b in rice phased small RNA biogenesis. Plant J. 2012;69(3): 462–74. doi: 10.1111/j.1365-313X.2011.04805.x 21973320 10.1111/j.1365-313X.2011.04805.x

[11] Komiya R, Ohyanagi H, Niihama M, Watanabe T, Nakano M, Kurata N, et al Rice germline-specific Argonaute MEL1 protein binds to phasiRNAs generated from more than 700 lincRNAs. Plant J. 2014;78(3): 385–97. doi: 10.1111/tpj.12483 24635777 10.1111/tpj.12483

[12] Zhai J, Zhang H, Arikit S, Huang K, Nan GL, Walbot V, et al Spatiotemporally dynamic, cell-type-dependent premeiotic and meiotic phasiRNAs in maize anthers. Proc Natl Acad Sci USA. 2015;112(10): 3146–51. doi: 10.1073/pnas.1418918112 25713378 10.1073/pnas.1418918112 PMC4364226

[13] Dukowic-Schulze S, Sundararajan A, Ramaraj T, Kianian S, Pawlowski WP, Mudge J, et al Novel meiotic miRNAs and indications for a role of phasiRNAs in meiosis. Front Plant Sci. 2016;7: 762 doi: 10.3389/fpls.2016.00762 27313591 10.3389/fpls.2016.00762 PMC4889585

[14] Fei Q, Xia R, Meyers BC. Phased, secondary, small interfering RNAs in posttranscriptional regulatory networks. Plant Cell. 2013;25(7): 2400–15 doi: 10.1105/tpc.113.114652 23881411 10.1105/tpc.113.114652 PMC3753373

[15] Allen E, Xie Z, Gustafson AM, Carrington JC. microRNA-directed phasing during *trans*-acting siRNA biogenesis in plants. Cell. 2005;121(2): 207–21. doi: 10.1016/j.cell.2005.04.004 15851028 10.1016/j.cell.2005.04.004

[16] Yoshikawa M, Peragine A, Park MY, Poethig RS. A pathway for the biogenesis of trans-acting siRNAs in *Arabidopsis*. Genes Dev. 2005;19(18): 2164–75. doi: 10.1101/gad.1352605 16131612 10.1101/gad.1352605 PMC1221887

[17] Rajagopalan R, Vaucheret H, Trejo J, Bartel DP. A diverse and evolutionarily fluid set of microRNAs in *Arabidopsis thaliana*. Genes Dev. 2006;20(24): 3407–25. doi: 10.1101/gad.1476406 17182867 10.1101/gad.1476406 PMC1698448

[18] Olmedo-Monfil V, Duran-Figueroa N, Arteaga-Vazquez M, Demesa-Arevalo E, Autran D, Grimanelli D, et al Control of female gamete formation by a small RNA pathway in *Arabidopsis*. Nature 2010;464(7288): 628–32. doi: 10.1038/nature08828 20208518 10.1038/nature08828 PMC4613780

[19] Slotkin RK, Vaughn M, Borges F, Tanurdzić M, Becker JD, Feijó JA, et al Epigenetic reprogramming and small RNA silencing of transposable elements in pollen. Cell. 2009;136(3): 461–72. doi: 10.1016/j.cell.2008.12.038 19203581 10.1016/j.cell.2008.12.038 PMC2661848

[20] Creasey KM, Zhai J, Borges F, Van Ex F, Regulski M, Meyers BC, et al miRNAs trigger widespread epigenetically activated siRNAs from transposons in *Arabidopsis*. Nature. 2014;508(7496): 411–5. doi: 10.1038/nature13069 24670663 10.1038/nature13069 PMC4074602

[21] Song X, Wang D, Ma L, Chen Z, Li P, Cui X, et al Rice RNA-dependent RNA polymerase 6 acts in small RNA biogenesis and spikelet development. Plant J. 2012;71(3): 378–89. doi: 10.1111/j.1365-313X.2012.05001.x 22443269 10.1111/j.1365-313X.2012.05001.x

[22] Goldberg RB, Beals TP, Sanders PM. Anther development: basic principles and practical applications. Plant Cell. 1993;5(10): 1217–29. doi: 10.1105/tpc.5.10.1217 8281038 10.1105/tpc.5.10.1217 PMC160355

[23] Yang WC, Ye D, Xu J, Sundaresan V. The *SPOROCYTELESS* gene of *Arabidopsis* is required for initiation of sporogenesis and encodes a novel nuclear protein. Genes Dev. 1999;13(16): 2108–17. 10465788 10.1101/gad.13.16.2108 PMC316961

[24] Nonomura KI, Miyoshi K, Eiguchi M, Suzuki T, Miyao A, Hirochika H, et al The *MSP1* gene is necessary to restrict the number of cells entering into male and female sporogenesis and to initiate anther wall formation in rice. Plant Cell. 2003;15(8): 1728–39. doi: 10.1105/tpc.012401 12897248 10.1105/tpc.012401 PMC167165

[25] Nan GL, Zhai J, Arikit S, Morrow D, Fernandes J, Mai L, et al MS23, a master basic helix-loop-helix factor, regulates the specification and development of the tapetum in maize. Development 2017; 144: 163–172. doi: 10.1242/dev.140673 27913638 10.1242/dev.140673

[26] Martínez G, Panda K, Köhler C, Slotkin RK. Silencing in sperm cells is directed by RNA movement from the surrounding nurse cell. Nat Plants. 2016 3 21;2:16030 doi: 10.1038/nplants.2016.30 27249563 10.1038/nplants.2016.30

[27] Fu Z, Yu J, Cheng X, Zong X, Xu J, Chen M et al The rice basic helix-loop-helix transcription factor TDR INTERACTING PROTEIN2 is a central switch in early anther development. Plant Cell. 2014;26(4): 1512–24. doi: 10.1105/tpc.114.123745 24755456 10.1105/tpc.114.123745 PMC4036568

[28] Ko SS, Li MJ, Sun-Ben KM, Ho YC, Lin YJ, Chuang MH, et al The bHLH142 transcription factor coordinates with TDR1 to modulate the expression of *EAT1* and regulate pollen development in rice. Plant Cell. 2014;26(6): 2486–504. doi: 10.1105/tpc.114.126292 24894043 10.1105/tpc.114.126292 PMC4114947

[29] Li N, Zhang DS, Liu HS, Yin CS, Li XX, Liang WQ, et al The rice tapetum degeneration retardation gene is required for tapetum degradation and anther development. Plant Cell. 2006;18(11): 2999–3014. doi: 10.1105/tpc.106.044107 17138695 10.1105/tpc.106.044107 PMC1693939

[30] Niu N, Liang W, Yang X, Jin W, Wilson ZA, Hu J, et al EAT1 promotes tapetal cell death by regulating aspartic proteases during male reproductive development in rice. Nat Commun. 2013;4: 1445 doi: 10.1038/ncomms2396 23385589 10.1038/ncomms2396

[31] Ji C, Li H, Chen L, Xie M, Wang F, Chen Y, et al A novel rice bHLH transcription factor, DTD, acts coordinately with TDR in controlling tapetum function and pollen development. Mol Plant. 2013;6(5): 1715–8. doi: 10.1093/mp/sst046 23519457 10.1093/mp/sst046

[32] Jung K.H., Han M.J., Lee Y.S., Kim Y.W., Hwang I., Kim M.J., et al Rice Undeveloped Tapetum1 is a major regulator of early tapetum development. Plant Cell. 2005;17(10): 2705–22. doi: 10.1105/tpc.105.034090 16141453 10.1105/tpc.105.034090 PMC1242267

[33] Du Z, Zhou X, Ling Y, Zhang Z, Su Z. agriGO: a GO analysis toolkit for the agricultural community. Nucleic Acids Res. 2010;38: W64–70. doi: 10.1093/nar/gkq310 20435677 10.1093/nar/gkq310 PMC2896167

[34] Zhang D, Shi J, Yang X. Role of lipid metabolism in plant pollen exine development. Subcell Biochem. 2016;86: 315–37. doi: 10.1007/978-3-319-25979-6_13 27023241 10.1007/978-3-319-25979-6_13

[35] Bailey TL, Boden M, Buske FA, Frith M, Grant CE, Clementi L, et al MEME SUITE: tools for motif discovery and searching. Nucleic Acid Res. 2009;37: W202–8. doi: 10.1093/nar/gkp335 19458158 10.1093/nar/gkp335 PMC2703892

[36] Chen HM, Chen LT, Patel K, Li YH, Baulcombe DC, Wu SH. 22-nucleotide RNAs trigger secondary siRNA biogenesis in plants. Proc Natl Acad Sci USA. 2010;107(34): 15269–74. doi: 10.1073/pnas.1001738107 20643946 10.1073/pnas.1001738107 PMC2930544

[37] Cuperus JT, Carbonell A, Fahlgren N, Garcia-Ruiz H, Burke RT, Takeda A, et al Unique functionality of 22-nt miRNAs in triggering RDR6-dependent siRNA biogenesis from target transcripts in *Arabidopsis*. Nat Struct Mol Biol. 2010;17(8): 997–1003. doi: 10.1038/nsmb.1866 20562854 10.1038/nsmb.1866 PMC2916640

[38] Zhou M. Gu L, Li P, Song X, Wei L, Chen Z, et al Degradome sequencing reveals endogenous small RNA targets in rice (*Oryza sativa* L. *ssp*. *indica*). Front Biol. 2010;5(1): 67–90. doi: 10.1007/s11515-010-0007-8

[39] Carretero-Paulet L, Galstyan A, Roig-Villanova I, Martinez-García JF, Bilbao-Castro JR, Robertson DL. Genome-wide classification and evolutionary analysis of the bHLH family of transcription factors in *Arabidopsis*, poplar, rice, moss, and algae. Plant Physiol. 2010;153(3): 1398–412. doi: 10.1104/pp.110.153593 20472752 10.1104/pp.110.153593 PMC2899937

[40] Wu L, Zhou H, Zhang Q, Zhang J, Ni F, Liu C, et al DNA methylation mediated by a microRNA pathway. Mol Cell. 2010;38(3): 465–75. doi: 10.1016/j.molcel.2010.03.008 20381393 10.1016/j.molcel.2010.03.008

[41] Wei L, Gu L, Song X, Cui X, Lu Z, Zhou M, et al Dicer-like 3 produces transposable element-associated 24-nt siRNAs that control agricultural traits in rice. Proc Natl Acad Sci U S A. 2014;111(10): 3877–82. doi: 10.1073/pnas.1318131111 24554078 10.1073/pnas.1318131111 PMC3956178

[42] Jones S. An overview of the basic helix-loop-helix proteins. Genome Biol. 2004;5(6):226 doi: 10.1186/gb-2004-5-6-226 15186484 10.1186/gb-2004-5-6-226 PMC463060

[43] Czech B, Hannon GJ. Small RNA sorting: matchmaking for Argonautes. Nat Rev Genet. 2011;12(1): 19–31. doi: 10.1038/nrg2916 21116305 10.1038/nrg2916 PMC3703915

[44] Wei W, Ba Z, Gao M, Wu Y, Ma Y, Amiard S, et al A role for small RNAs in DNA double-strand break repair. Cell. 2012;149(1):101–12. doi: 10.1016/j.cell.2012.03.002 22445173 10.1016/j.cell.2012.03.002

[45] Miki D, Zhu P, Zhang W, Mao Y, Feng Z, Huang H, et al Efficient Generation of diRNAs Requires Components in the Posttranscriptional Gene Silencing Pathway. Sci Rep. 2017;7(1):301 doi: 10.1038/s41598-017-00374-7 28331197 10.1038/s41598-017-00374-7 PMC5428250

[46] Liu H, Nonomura KI. A wide reprogramming of histone H3 modifications during male meiosis I in rice is dependent on the Argonaute protein MEL1. J Cell Sci. 2016;129(19): 3553–61. doi: 10.1242/jcs.184937 27521428 10.1242/jcs.184937

[47] Zhao X, de Palma J, Oane R, Gamuyao R, Luo M, Chaudhury A, et al OsTDL1A binds to the LRR domain of rice receptor kinase MSP1, and is required to limit sporocyte numbers. Plant J. 2008 54(3): 375–87. doi: 10.1111/j.1365-313X.2008.03426.x 18248596 10.1111/j.1365-313X.2008.03426.x PMC2408674

[48] Melnyk CW, Molnar A, Baulcombe DC. Intercellular and systemic movement of RNA silencing signals. EMBO J. 2011; 30 (17): 3553–3563. doi: 10.1038/emboj.2011.274 21878996 10.1038/emboj.2011.274 PMC3181474

[49] Ünal M, Vardar F, Aytürk Ö. Callose in plant sexual reproduction In: Silva-Opps, the editor. Cytoplasmic connexions between angiosperm meiocytes. Current progress in biological research, InTech; 2013 pp. 319–343. doi: 10.5772/53001

[50] Heslop-Harrison J. Cytoplasmic connexions between angiosperm meiocytes. Ann Bot. 1966;30(2): 221–30. doi: 10.1093/oxfordjournals.aob.a084069

[51] Fei Q, Yang L, Liang W, Zhang D, Meyers BC. Dynamic changes of small RNAs in rice spikelet development reveal specialized reproductive phasiRNA pathways. J Exp Bot. 2016;67(21): 6037–49. doi: 10.1093/jxb/erw361 27702997 10.1093/jxb/erw361 PMC5100018

[52] Oliver C, Santos JL, Pradillo M. Accurate chromosome segregation at first meiotic division requires AGO4, a protein involved in RNA-directed DNA methylation in *Arabidopsis thaliana*. Genetics. 2016;204(2): 543–53. doi: 10.1534/genetics.116.189217 27466226 10.1534/genetics.116.189217 PMC5068845

[53] Singh M, Goel S, Meeley RB, Dantec C, Parrinello H, Michaud C, et al Production of viable gametes without meiosis in maize deficient for an ARGONAUTE protein. Plant Cell. 2011;23(2):443–58. doi: 10.1105/tpc.110.079020 21325139 10.1105/tpc.110.079020 PMC3077773

[54] Pontes O, Li CF, Costa Nunes P, Haag J, Ream T, Vitins A, et al The *Arabidopsis* chromatin-modifying nuclear siRNA pathway involves a nucleolar RNA processing center. Cell. 2006;126(1): 79–92. doi: 10.1016/j.cell.2006.05.031 16839878 10.1016/j.cell.2006.05.031

[55] Li CF, Pontes O, El-Shami M, Henderson IR, Bernatavichute YV, Chan SW, et al An ARGONAUTE4-containing nuclear processing center colocalized with Cajal bodies in *Arabidopsis thaliana*. Cell. 2006;126(1): 93–106. doi: 10.1016/j.cell.2006.05.032 16839879 10.1016/j.cell.2006.05.032

[56] Chan SW. Inputs and outputs for chromatin-targeted RNAi. Trends Plant Sci. 2008;13(7): 383–389. doi: 10.1016/j.tplants.2008.05.001 18550415 10.1016/j.tplants.2008.05.001

[57] He XJ, Hsu YF, Zhu S, Wierzbicki AT, Pontes O, Pikaard CS, et al An effector of RNA-directed DNA methylation in *Arabidopsis* is an ARGONAUTE 4- and RNA-binding protein. Cell. 2009;137(3): 498–508. doi: 10.1016/j.cell.2009.04.028 19410546 10.1016/j.cell.2009.04.028 PMC2700824

[58] Matzke M, Kanno T, Daxinger L, Huettel B, Matzke AJ. RNA-mediated chromatin-based silencing in plants. Curr Opin Cell Biol. 2009;21(3):367–76. doi: 10.1016/j.ceb.2009.01.025 19243928 10.1016/j.ceb.2009.01.025

[59] Numa H, Kim JM, Matsui A, Kurihara Y, Morosawa T, Ishida J, et al Transduction of RNA-directed DNA methylation signals to repressive histone marks in *Arabidopsis thaliana*. EMBO J. 2010;29(2): 352–62. doi: 10.1038/emboj.2009.374 20010696 10.1038/emboj.2009.374 PMC2824457

[60] Hirochika H, Sugimoto K, Otsuki Y, Tsugawa H, Kanda M. Retrotransposon of rice involved in mutations induced by tissue culture. Proc Natl Acad Sci USA. 1996;93(15): 7783–8. 8755553 10.1073/pnas.93.15.7783 PMC38825

[61] Jeon JS, Lee S, Jung KH, Jun SH, Jeong DH, Lee J, et al T-DNA insertional mutagenesis for functional genomics in rice. Plant J. 2000;22(6): 561–70. doi: 10.1046/j.1365-313x.2000.00767.x 10886776 10.1046/j.1365-313x.2000.00767.x

[62] Jeong DH, An S, Park S, Kang HG, Park GG, Kim SR, et al Generation of a flanking sequence-tag database for activation-tagging lines in japonica rice. Plant J 2006;45(1): 123–32. doi: 10.1111/j.1365-313X.2005.02610.x 16367959 10.1111/j.1365-313X.2005.02610.x

[63] Matsuo Y, Arimura S, Tsutsumi N. Distribution of cellulosic wall in the anthers of *Arabidopsis* during microsporogenesis. Plant Cell Rep. 2013;32(11): 1743–50. doi: 10.1007/s00299-013-1487-1 23893118 10.1007/s00299-013-1487-1

[64] Chiu WI, Niwa Y, Zeng W, Hirano T, Kobayashi H, Sheen J. Engineered GFP as a vital reporter in plants. Curr Biol. 1996;6(3): 325–30. 8805250 10.1016/s0960-9822(02)00483-9

[65] Fuse T, Sasaki T, Yano M. Ti-plasmid vectors useful for functional analysis of rice genes. Plant Biotechnol. 2001;18: 219–222.

[66] Nakamura S, Mano S, Tanaka Y, Ohnishi M, Nakamori C, Araki M, et al Gateway binary vectors with the bialaphos resistance gene, *bar*, as a selection marker for plant transformation. Biosci Biotechnol Biochem. 2010;74(6): 1315–19. doi: 10.1271/bbb.100184 20530878 10.1271/bbb.100184

[67] Toki S, Hara N, Ono K, Onodera H, Tagiri A, Oka S, et al Early infection of scutellum tissue with *Agrobacterium* allows high-speed transformation of rice. Plant J. 2006 47(6): 969–76. doi: 10.1111/j.1365-313X.2006.02836.x 16961734 10.1111/j.1365-313X.2006.02836.x

[68] Gaidatzis D, Lerch A, Hahne F, Stadler MB. QuasR: quantification and annotation of short reads in R. Bioinformatics. 2015;31: 1130–2. doi: 10.1093/bioinformatics/btu781 25417205 10.1093/bioinformatics/btu781 PMC4382904

[69] Trapnell C, Pachter L, Salzberg SL. TopHat: discovering splice junctions with RNA-Seq. Bioinformatics. 2009;25(9): 1105–11. doi: 10.1093/bioinformatics/btp120 19289445 10.1093/bioinformatics/btp120 PMC2672628

[70] Trapnell C, Hendrickson DG, Sauvageau M, Goff L, Rinn JL, Pacher L. Differential analysis of gene regulation at transcript resolution with RNA-seq. Nat Biotechnol. 2013;31(1): 46–53. doi: 10.1038/nbt.2450 23222703 10.1038/nbt.2450 PMC3869392

[71] Quinlan AR, Hall IM. BEDTools: a flexible suite of utilities for comparing genomic features. Bioinformatics. 2010;26(6): 841–2. doi: 10.1093/bioinformatics/btq033 20110278 10.1093/bioinformatics/btq033 PMC2832824

[72] Morgan M, Anders S, Lawrence M, Aboyoun P, Pagès H, Gentleman R. ShortRead: a bioconductor package for input, quality assessment and exploration of high-throughput sequence data. Bioinformatics. 2009;25(19): 2607–08. doi: 10.1093/bioinformatics/btp450 19654119 10.1093/bioinformatics/btp450 PMC2752612

[73] Howell MD, Fahlgren N, Chapman EJ, Cumbie JS, Sullivan CM, Givan SA, et al Genome-wide analysis of the RNA-DEPENDENT RNA POLYMERASE6/DICER-LIKE4 pathway in *Arabidopsis* reveals dependency on miRNA- and tasiRNA-directed targeting. Plant Cell. 2007;19(3): 926–42. doi: 10.1105/tpc.107.050062 17400893 10.1105/tpc.107.050062 PMC1867363

[74] Langmead B, Salzberg SL. Fast gapped-read alignment with Bowtie 2. Nat Methods. 2012;9(4):357–9. doi: 10.1038/nmeth.1923 22388286 10.1038/nmeth.1923 PMC3322381

[75] Tsuda K, Ito Y, Sato Y, Kurata N. Positive autoregulation of a *KNOX* gene is essential for shoot apical meristem maintenance in rice. Plant Cell. 2011;23(12): 4368–81. doi: 10.1105/tpc.111.090050 22207572 10.1105/tpc.111.090050 PMC3269871

[76] Zhang Y, Su J, Duan S, Ao Y, Dai J, Liu J, et al A highly efficient rice green tissue protoplast system for transient gene expression and studying light/chloroplast-related processes. Plant Methods. 2011;7(1):30 doi: 10.1186/1746-4811-7-30 21961694 10.1186/1746-4811-7-30 PMC3203094

[77] Tsugama D, Liu H, Liu S, Takano T. *Arabidopsis* heterotrimeric G protein β subunit interacts with a plasma membrane 2C-type protein phosphatase, PP2C52. Biochim Biophys Acta. 2012;1823(12):2254–60. doi: 10.1016/j.bbamcr.2012.10.001 23058975 10.1016/j.bbamcr.2012.10.001

[78] Robinson JT, Thorvaldsdóttir H, Winckler W, Guttman M, Lander ES, Getz G, et al Integrative genomics viewer. Nat Biotechnol. 2011;29(1):24–6. doi: 10.1038/nbt.1754 21221095 10.1038/nbt.1754 PMC3346182

