# Correction: EAT1 transcription factor, a non-cell-autonomous regulator of pollen production, activates meiotic small RNA biogenesis in rice anther tapetum

**DOI:** 10.1371/journal.pgen.1008033

**Published:** 2019-03-06

**Authors:** Seijiro Ono, Hua Liu, Katsutoshi Tsuda, Eigo Fukai, Keisuke Tanaka, Takuji Sasaki, Ken-Ichi Nonomura

There are errors in the sample titles contained within S8 Table. Please view the corrected version below.

## Supporting information

S8 Table Identifiers of mRNAseq, sRNAseq and MEL1-RIPseq data deposited to DDBJ. (XLSX)
