# Supplementary figures and images for: EAT1 transcription factor, a non-cell-autonomous regulator of pollen production, activates meiotic small RNA biogenesis in rice anther tapetum
Source: PLoS Genet. 2018 Feb 12;14(2):e1007238. doi: 10.1371/journal.pgen.1007238 (PMC5825165; doi:10.1371/journal.pgen.1007238)

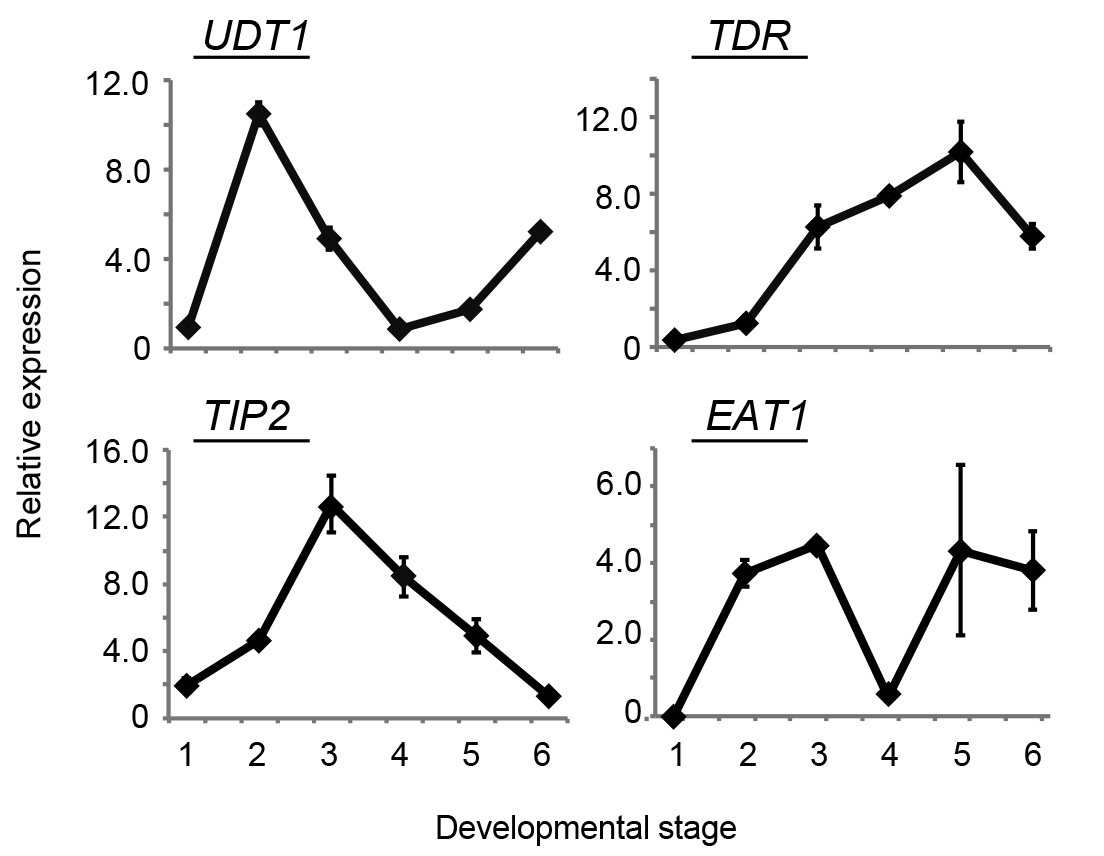

Supplement: S1 Fig — Expression patterns of four tapetum-related bHLH genes; UDT1, TDR, TIP2 and EAT1, in wild-type (cv. Nipponbare) anther development. The bottom numbers of the developmental stage correspond to Table 1. Relative expression values and standard errors were calculated usng three biological replicates. (TIF) [file pgen.1007238.s001.tif]

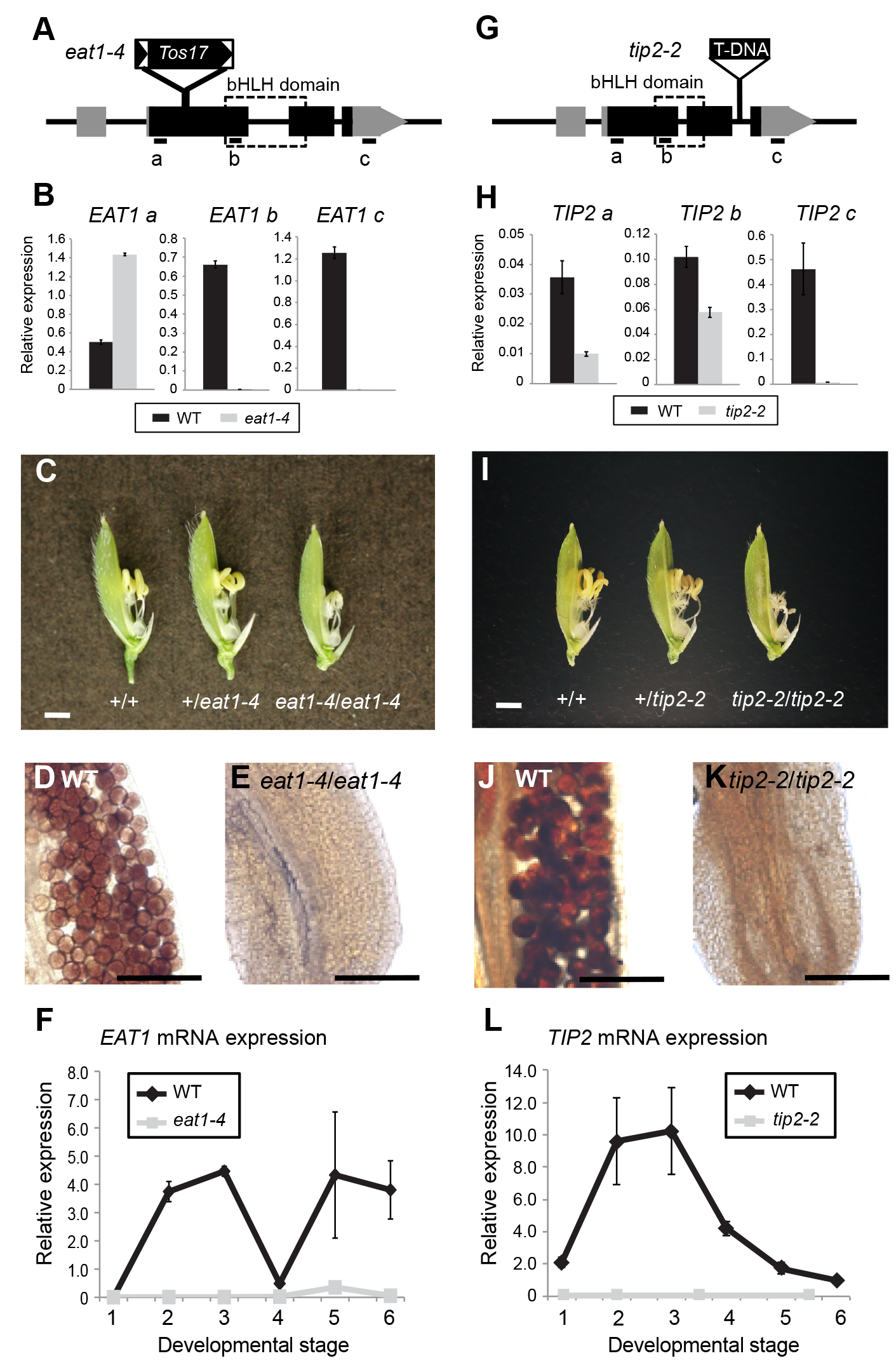

Supplement: S2 Fig — (A and G) Genomic structure of EAT1 and Tos17 insertion of eat1-4 (A) and TIP2 and T-DNA insertion of tip2-2 (G). (B and H) qRT-PCR results of underlined regions of EAT1 transcript in wild-type and eat1-4 flowers (B) and of TIP2 transcript in wild-type and tip2-2 flowers (H). In (B) and (H), total RNAs from early meiotic flowers (around 2.0 mm) long were used. (C and I) Flower morphology of eat1-4 (C) and tip2-2 (I). Bars, 1 mm. (D, E, J and K) I2KI staining of mature pollen in the anther of EAT1 wild type (D), eat1-4 mutant (E), TIP2 wild type (J), and tip2-2 mutant (K). Bars, 100 μm. (F and L) EAT1 mRNA expression during anther development in wild type (cv. Nipponbare) and eat1-4 plants (F) and TIP2 mRNA expression in wild type (cv. Dongjin) and tip2-2 anthers (L). In (F), expression data of EAT1 transcripts in wild-type anthers were identical to those of S1 Fig. In qRT-PCR analyses, relative expression values and standard errors were calculated by three biological replicates. (TIF) [file pgen.1007238.s002.tif]

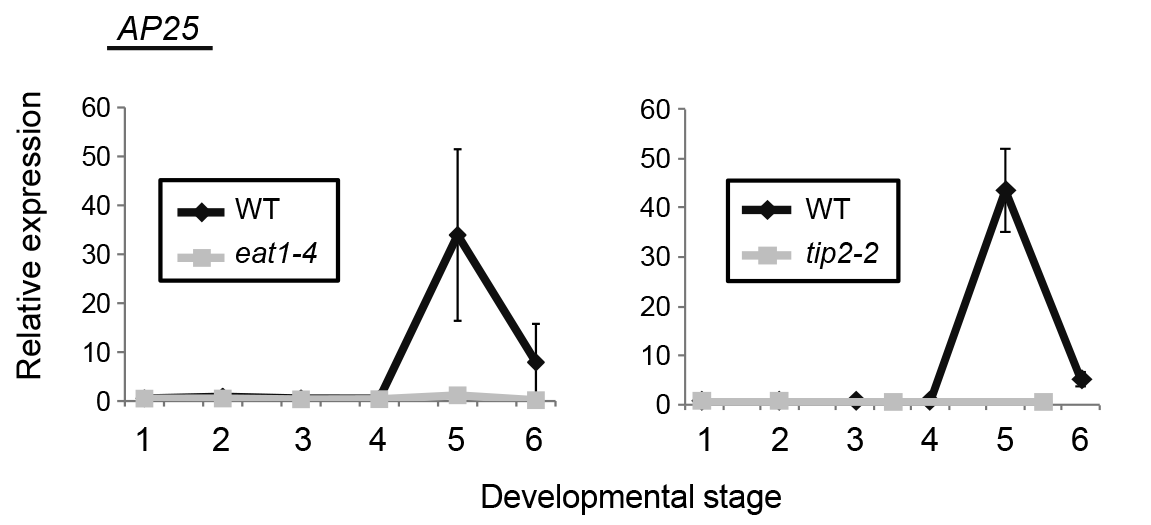

Supplement: S3 Fig — AP25 expression during anther development in eat1-4 (left), tip2-2 (right) and their respective wild-type siblings. Relative expression values and standard errors were calculated by three biological replicates. (TIF) [file pgen.1007238.s003.tif]

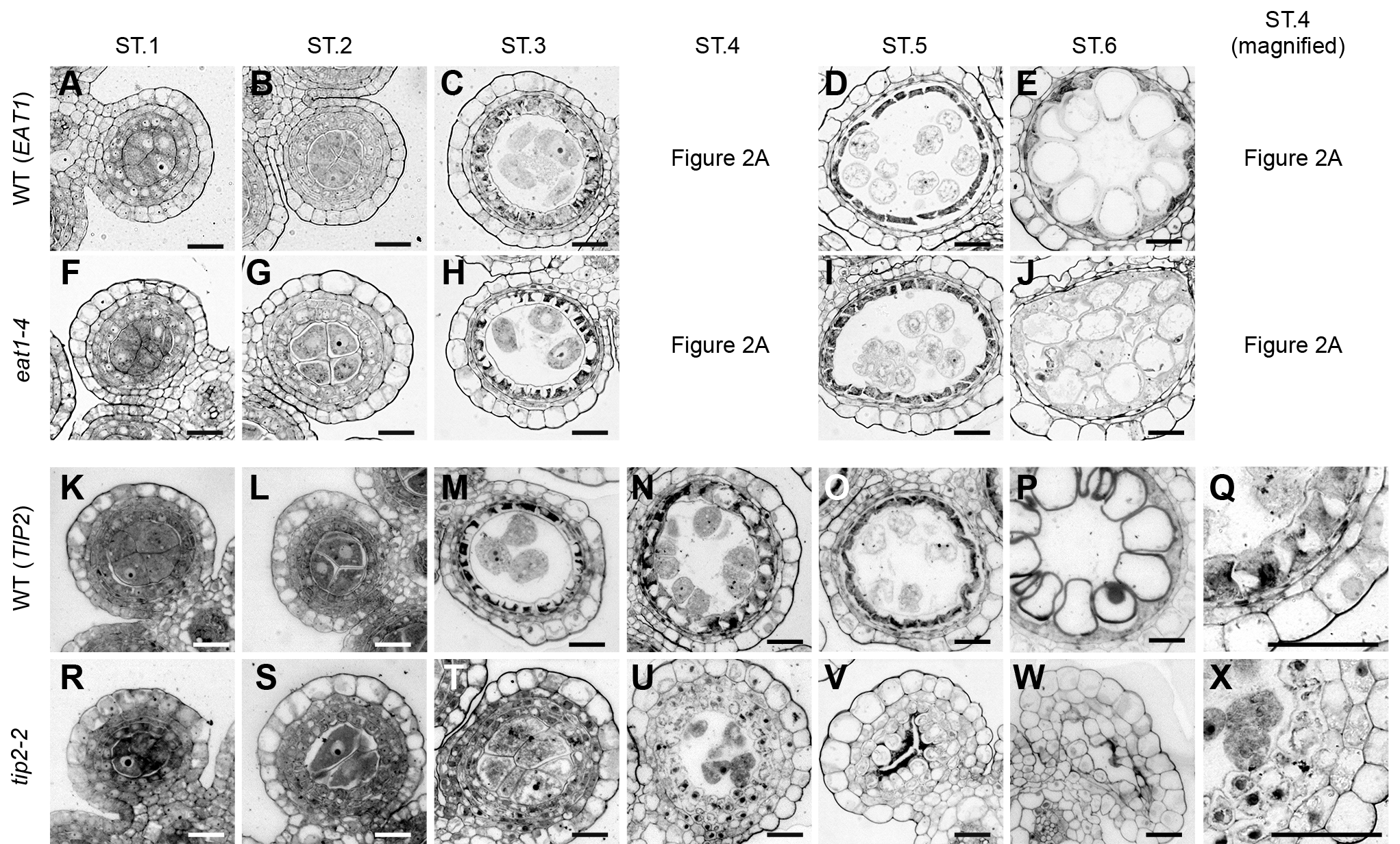

Supplement: S4 Fig — (A to J) Anther cross-sections of wild-type (EAT1) (A to E) and eat1-4 (F to J). The cross-sections of ST.4 and their magnified views are shown in Fig 2A. (K to X) Anther cross-sections of wild-type (TIP2) (K to Q) and tip2-2 (R to X). (A, F, K, and R): ST.1; (B, G, L and S): ST.2; (C, H, M, and T): ST.3; (N and U): ST.4; (D, I, O and V): ST.5; (E, J, P and W): ST.6; (Q and X): magnified view of ST.4. Bars, 20 μm. (TIF) [file pgen.1007238.s004.tif]

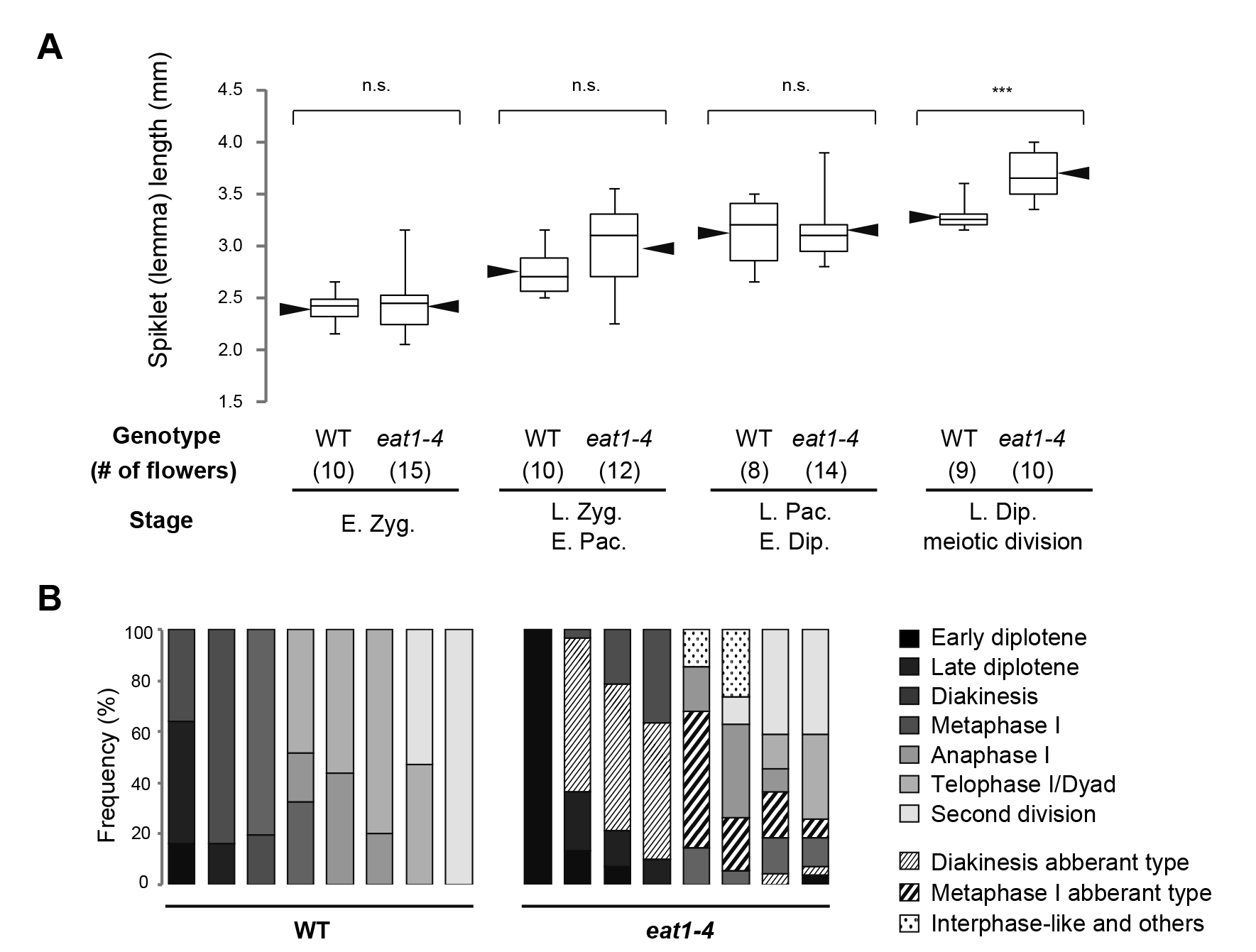

Supplement: S5 Fig — (A) Box plots of spikelet lengths in each meiosis I stage in wild-type and eat1-4 mutant anthers. Zyg.; early zygotene, Pac.; pachytene, Dip.; diplotene, Div; the stage including diakinesis, metaphase I, anaphase I, dyad, and second division. n.s. and *** indicate no significance and significance at P = 0.001 (Student's t-test), respectively, between the wild-type and eat1-4. Arrowheads indicate average values. (B) Column charts showing the spectrum of meiotic stages in single wild-type and eat1-4 anthers. (TIF) [file pgen.1007238.s005.tif]

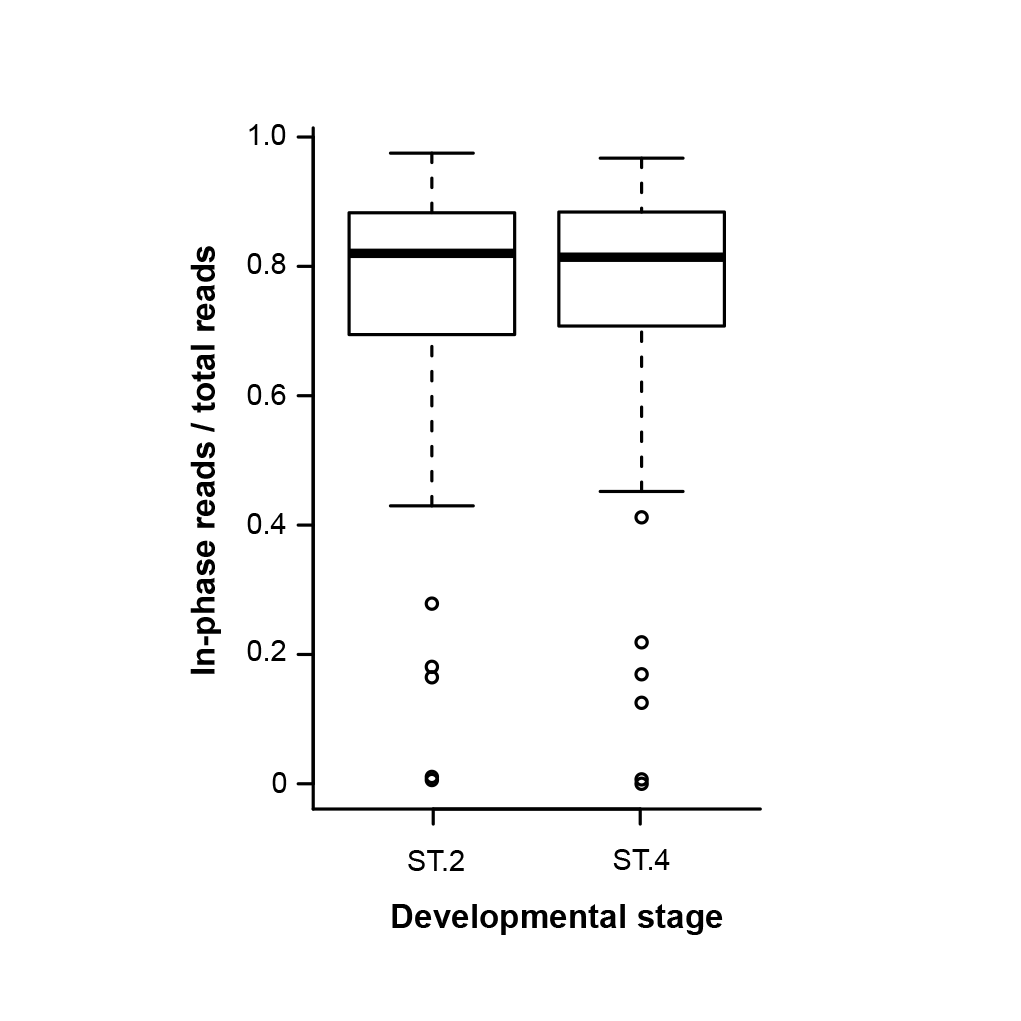

Supplement: S6 Fig — Of 254,163 and 877,203 reads of 24-nt small RNAs from ST.2 and ST. 4 anthers, 329,112 (ST.2) and 1,138,234 reads (ST.4) were defined as in-phase reads for the 24-nt phased interval that starts from the predicted miR2275 cleavage site (Fig 4B and 4C) on 93 24-PHAS loci identified in this study. Then, the frequency of in-phase reads to total reads were box-plotted. The sRNA-seq reads from three replicates were combined in each stage and plotted. The median values were 0.814 and 0.820 in wild-type ST.2 and ST.4 samples, respectively. (TIF) [file pgen.1007238.s006.tif]

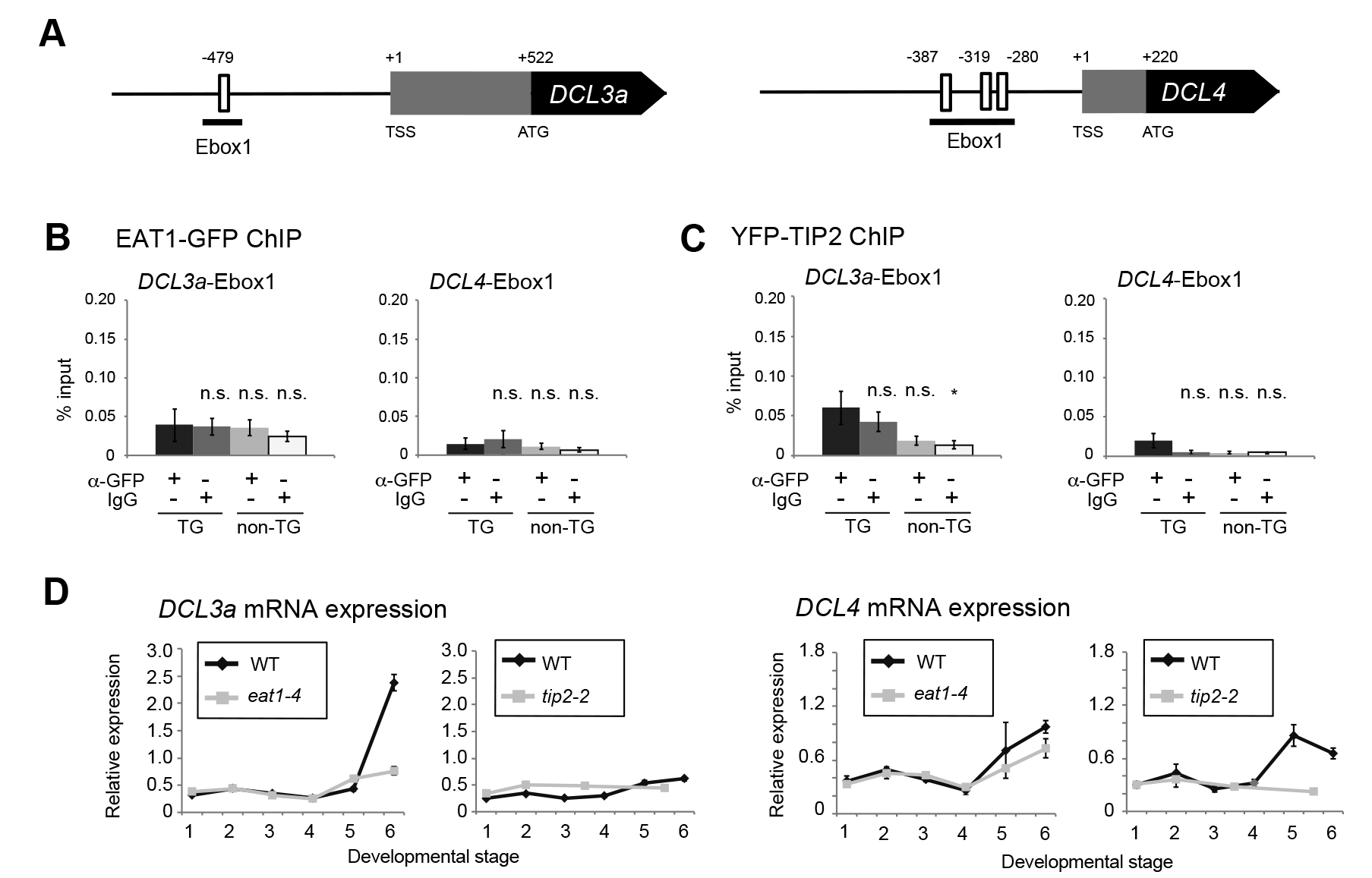

Supplement: S7 Fig — (A) Structure of 5ʹ upstream regions of DCL3a and DCL4. The diagrams are equivalent to Fig 5D. (B and C) ChIP-qPCR results of DCL3a and DCL4 promoter region using transgenic (TG) plants expressing EAT1-GFP (B) and YFP-TIP2 (C). n.s.; not significant. * and **; significant at P = 0.05 and P = 0.01 in Student's t-test, respectively, less than the leftmost positive ChIP result in each graph. Relative abundance and standard errors were calculated by two or three biological replicates each subjected to three PCR replications. (D) qRT-PCR results of DCL3a and DCL4 during anther development of eat1-4, tip2-2 and their respective wild-type siblings. Relative expression values and standard errors were calculated by three biological replicates. (TIF) [file pgen.1007238.s007.tif]

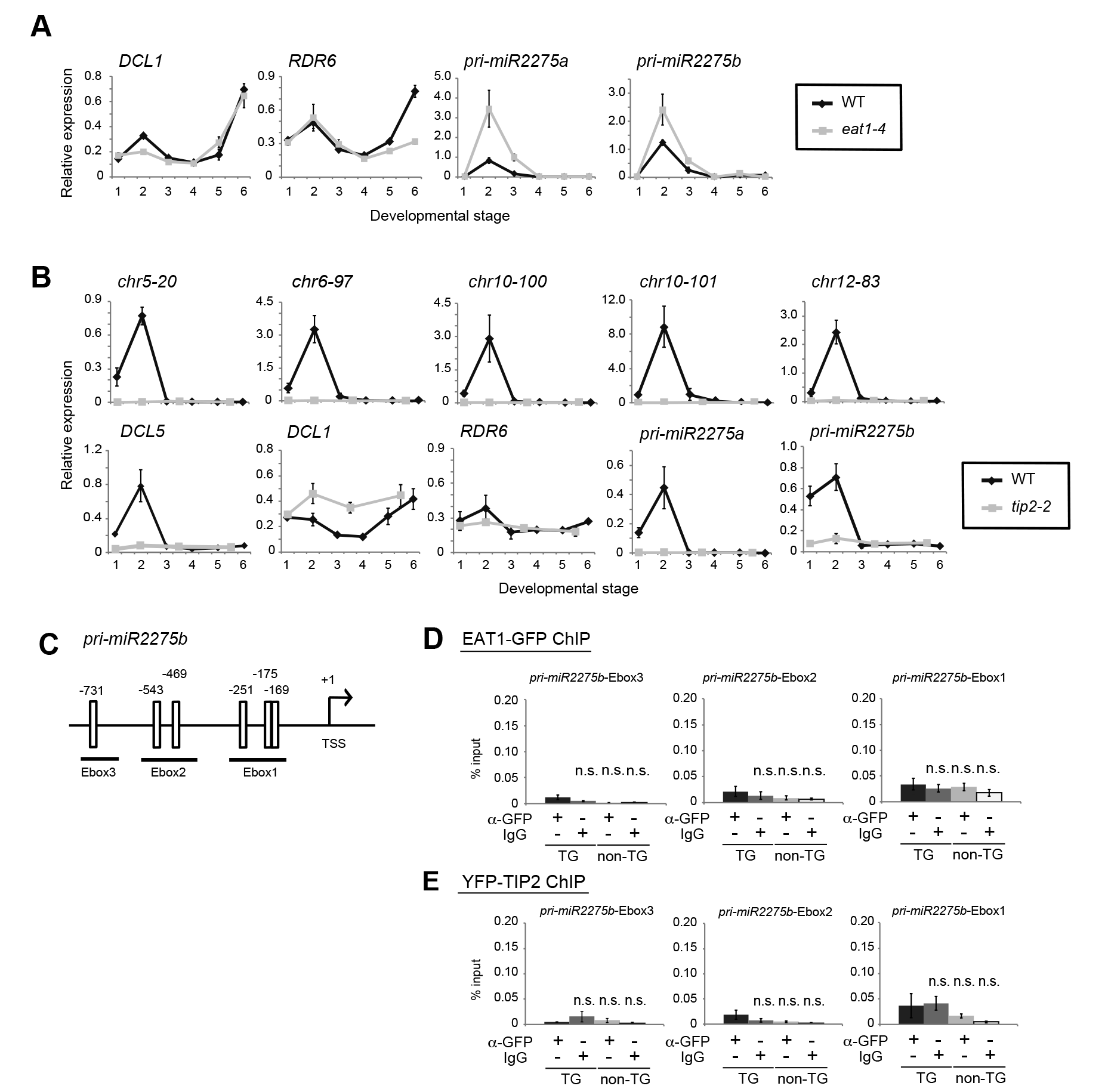

Supplement: S8 Fig — (A) qRT-PCR results of 24-nt phasiRNA biogenesis-related genes, DCL1, RDR6, and two pri-miR2275 transcripts in wild-type and eat1-4 anthers. (B) qRT-PCR results of five 24-PHAS transcripts, DCL5, DCL1, RDR6, and two pri-miR2275 transcripts in wild-type and tip2-2 anthers. In qRT-PCR analyses, relative expression values and standard errors were calculated by three biological replicates. (C) Schematic illustration of genomic compositions of the 5ʹ upstream regions of pri-miR2275b locus. The diagrams are equivalent to Fig 5A. (D and E) ChIP-qPCR results of pri-miR2275b promoters using TG plants expressing EAT1-GFP (E) and YFP-TIP2 (E). In ChIP-qPCR analyses, relative abundance and standard errors were calculated by two or three biological replicates each subjected to three PCR replications. n.s.; not significant. * and **; significant at P = 0.05 and P = 0.01 in Student's t-test, respectively, less than the leftmost positive ChIP result in each graph. (TIF) [file pgen.1007238.s008.tif]

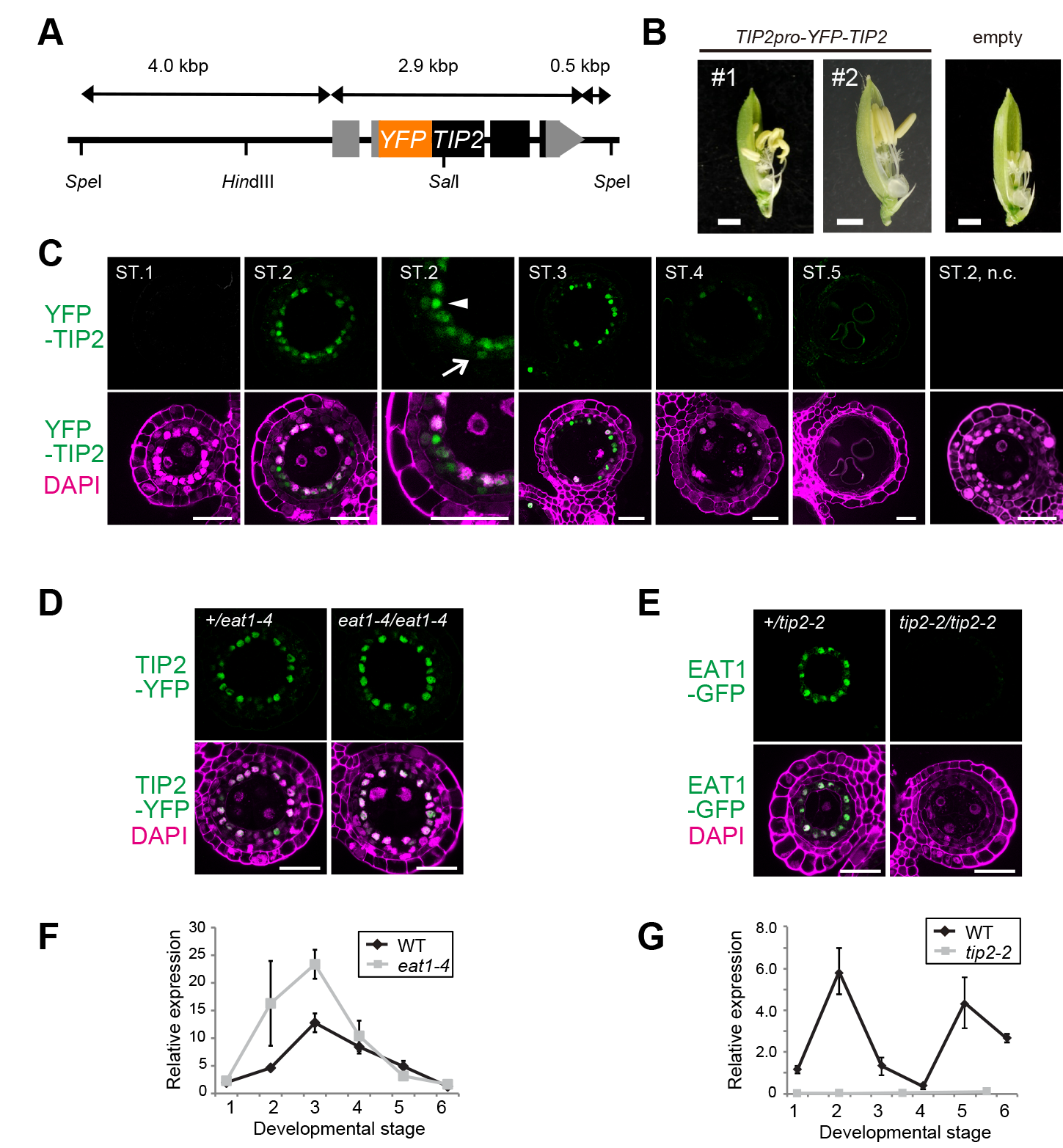

Supplement: S9 Fig — (A) Diagram of the TIP2pro-YFP-TIP2 transcriptional fusion construct. Closed and grey boxes indicate protein coding and untranslated regions, respectively. (B) tip2-2/tip2-2 flowers of T0 plants carrying EAT1pro-EAT1-GFP (#1, #2) and an empty vector. Bars, 1 mm. (C) YFP-TIP2 signals (green) in developing anther sections from ST.1 to ST.5. in a transgenic plant harboring TIP2pro-YFP-TIP2. YFP-TIP2 signals were intensified in tapetal nuclei (arrowhead) and also detected in middle layer nuclei (arrow) in ST.2 and ST. 3 anthers, and not detected in the ST.2 anther from the negative control (n.c., right most panel). Bars, 20 μm. (D and E) TIP2-YFP expression and localization in wild-type and eat1-4 ST.2 anthers (D) and EAT1-GFP expression and localization in wild-type and tip2-2 ST.2 anthers (E). Bars, 20 μm. (F and G) Expression pattern of TIP2 mRNA in wild-type and eat1-4 anthers (F), and EAT1 mRNA in wild-type and tip2-2 anthers (G). Relative expression values and standard errors were calculated by three biological replicates. (TIF) [file pgen.1007238.s009.tif]

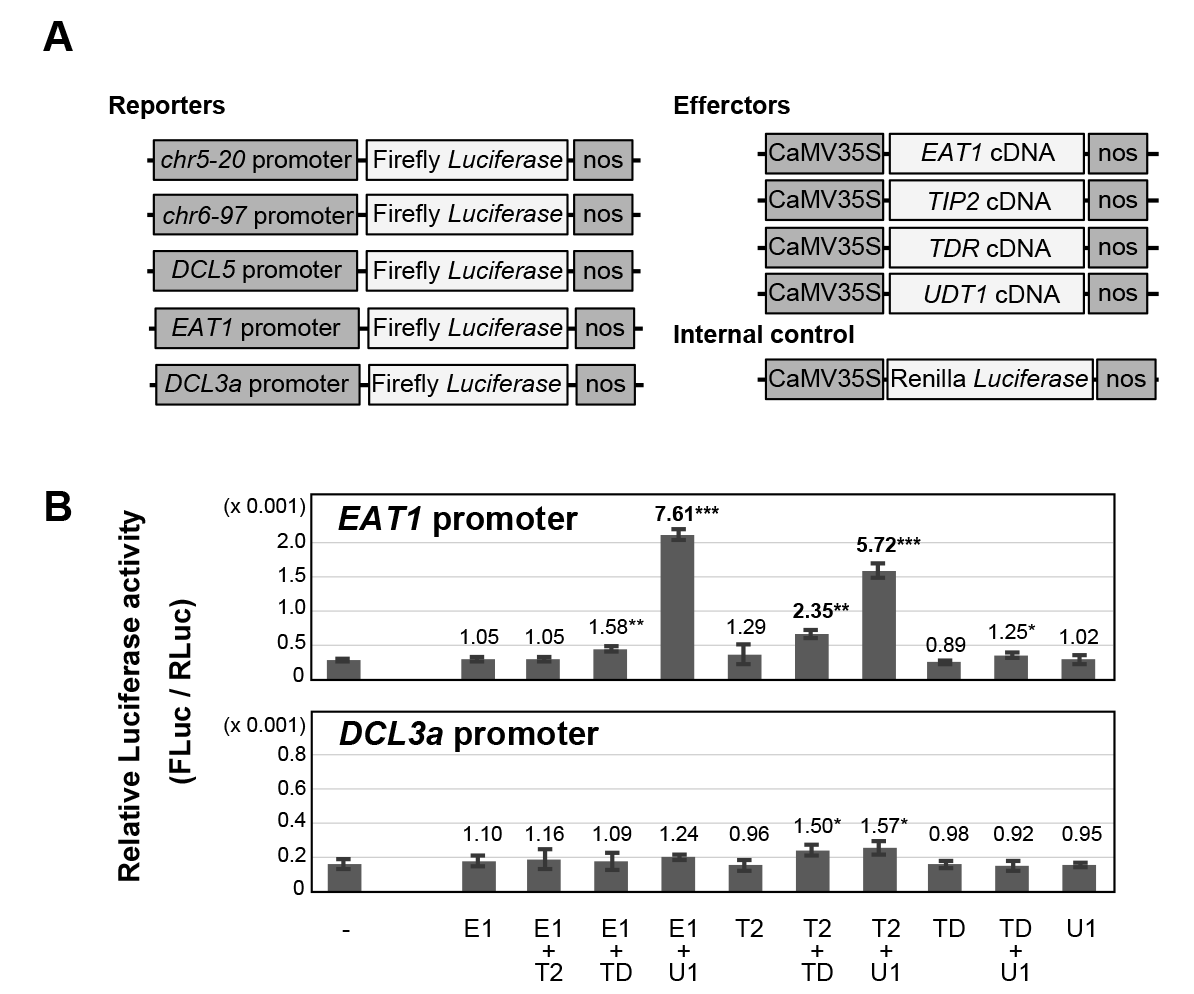

Supplement: S10 Fig — (A) Schematic diagrams of the reporter, effector and internal control constructs used in the transient expression assay. The reporter carries a 2-kbp promoter region of the 24-PHASs (chr5-20, chr6-97), DCL5, EAT1 or DCL3a fused with the firefly Luciferase. CaMV35S; cauliflower mosaic virus 35S promoter, nos; nopaline synthase terminator. (B) The results of the transient expression assay. Any one or two effector plasmids encoding EAT1 (E1), TIP2 (T2), UDT1 (U1) and TDR (TD) proteins were cotransfected with the reporter constructs into rice protoplasts. The number above each bar is the fold change of the Luciferase activity compared to the negative control without the effector (leftmost bars). *, ** and ***; the significant fold changes at P = 0.05, 0.01 and 0.001 in Student’s t-test, respectively, compared to the negative control. Error bars indicated standard deviation of three biological replicates. The significant >2 fold changes were in bold. (TIF) [file pgen.1007238.s010.tif]

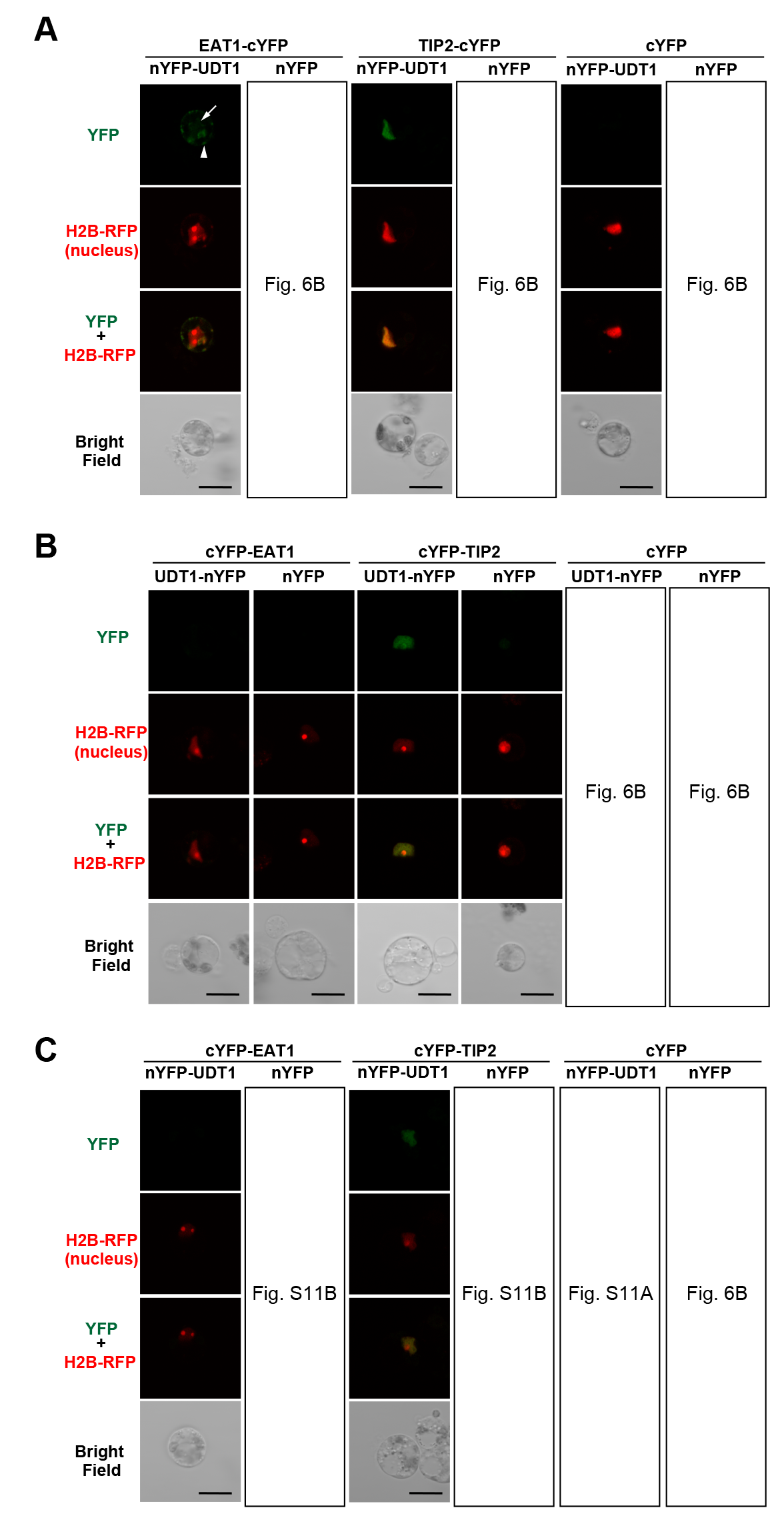

Supplement: S11 Fig — (A) BiFC results of EAT1-cYFP and TIP2-cYFP constructs combined with a nYFP-UDT1 sonstruct and those of negative control combinations. (B) BiFC results of cYFP-EAT1 and cYFP-TIP2 constructs combined with a UDT1-nYFP construct and those of negative control combinations. (C) BiFC results of cYFP-EAT1 and cYFP-TIP2 constructs combined with a nYFP-UDT1 construct. Some negative control results common in Fig 6B and S11 Fig. were indicated by empty boxes. (TIF) [file pgen.1007238.s011.tif]

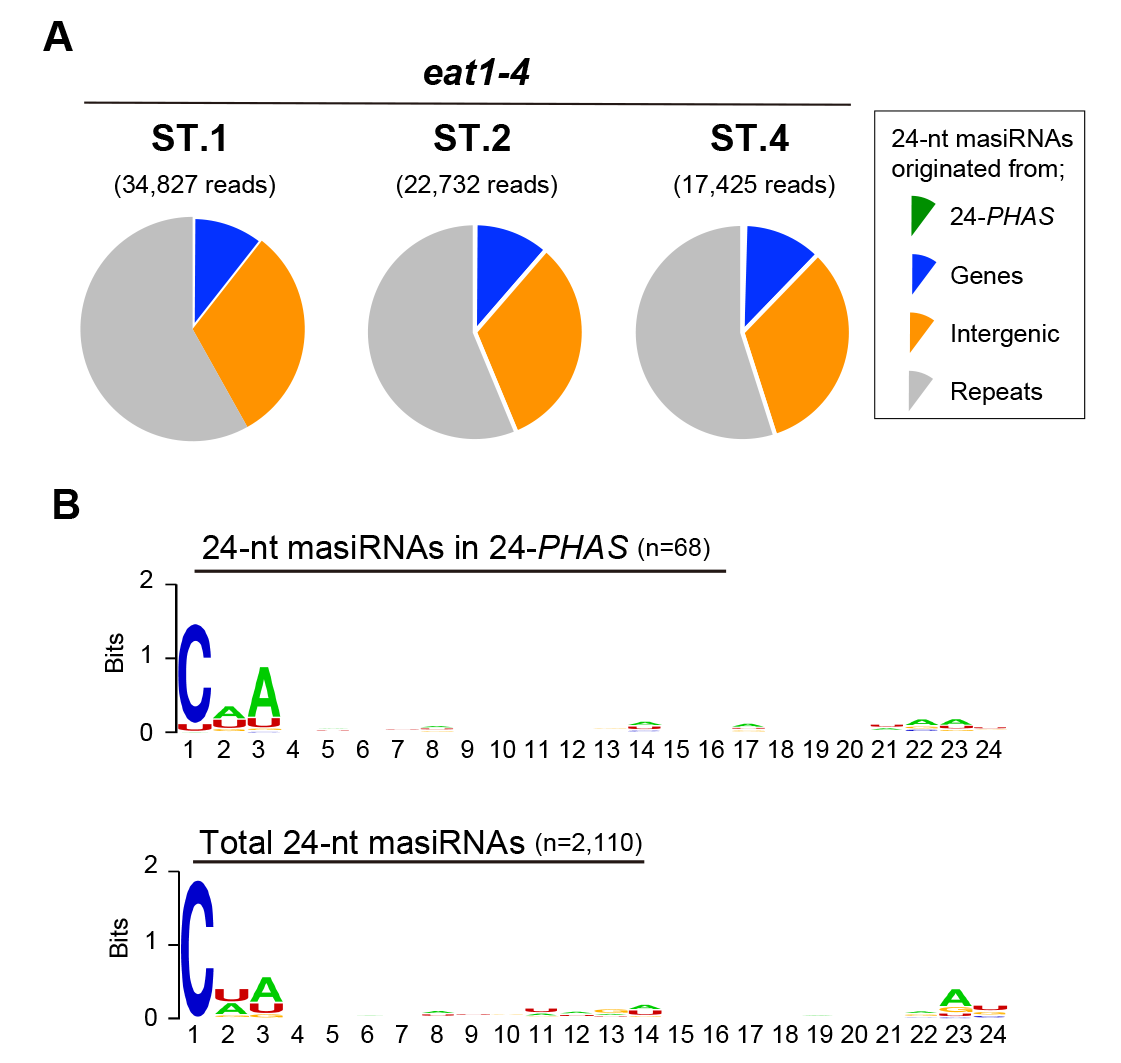

Supplement: S12 Fig — (A) Pie-charts representing the ratios of 24-nt MEL1-associating siRNAs (masiRNAs) originated from 24-PHAS loci, protein-coding genes, intergenic regions except for 24-PHAS loci and repetitive regions, in eat1-4 anthers at ST.1, ST.2 and ST.4 stages. The numbers with parentheses indicated the read counts of 24-nt masiRNAs extracted from MEL1-IPseq results. (B) The sequence logos generated from 68 species of 24-nt masiRNAs mapped onto 24-PHAS loci (top), and from all 2,110 species of 24-nt masiRNAs mapped on the rice genome (bottom). (TIF) [file pgen.1007238.s012.tif]

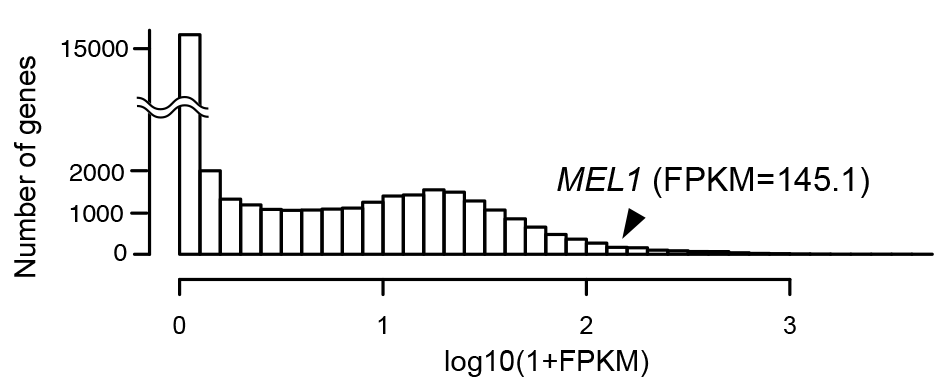

Supplement: S13 Fig — A histogram representing the distribution of FPKM values of all 38,311 rice genes in wild-type ST.2 anthers. The area where MEL1 included was indicated by an arrowhead. (TIF) [file pgen.1007238.s013.tif]
