# Supplementary material for: EAT1 transcription factor, a non-cell-autonomous regulator of pollen production, activates meiotic small RNA biogenesis in rice anther tapetum
Source: PLoS Genet. 2018 Feb 12;14(2):e1007238. doi: 10.1371/journal.pgen.1007238 (PMC5825165; doi:10.1371/journal.pgen.1007238)
Supplement: S7 Table — (DOCX) [file pgen.1007238.s021.docx]

**S7 Table. Anther length and corresponding developmental stages in *eat1-4* and *tip2-2* plants.**

| Stages | Anther length  (*EAT1* wild-type) | Anther length  (*eat1-4*) | Anther length  (*TIP2* wild-type) | Anther length  (*tip2-2*) |
| --- | --- | --- | --- | --- |
| ST.1 | 0.3-0.4 mm | 0.3-0.4 mm | 0.3-0.4 mm | 0.3-0.4 mm |
| ST.2 | 0.4-0.5 mm | 0.4-0.5 mm | 0.4-0.5 mm | 0.4-0.5 mm |
| ST.3 | 0.6-0.7 mm | 0.6-0.7 mm | 0.6-0.7 mm | 0.6-0.7 mm ^a^ |
| ST.4 | 0.8-0.9 mm | 0.8-0.9 mm | 0.7-0.8 mm |  |
| ST.5 | 1.0-1.5 mm | 1.0-1.2 mm | 0.9-1.0 mm | 0.8-0.9 mm ^a^ |
| ST.6 | >1.5 mm | >1.2 mm | >1.0 mm |  |

^a^ These anthers were collected as *tip2-2* ST.3-4 and *tip2-2* ST.5-6, respectively.
